# Genomic Analysis of the Columbian Plumage Pattern in Various Chicken Breeds

**DOI:** 10.3390/ani16142153

**Published:** 2026-07-11

**Authors:** Anastasiia I. Azovtseva, Anna E. Ryabova, Yuri S. Shcherbakov, Tatiana A. Larkina, Anatoly B. Vakhrameev, Natalia V. Dementieva

**Affiliations:** Russian Research Institute of Farm Animal Genetics and Breeding (RRIFAGB)—Branch of the L.K. Ernst, Federal Science Centre for Animal Husbandry, Pushkin, 196601 St. Petersburg, Russia; aniuta.riabova2016@yandex.ru (A.E.R.); yura.10.08.94.94@mail.ru (Y.S.S.); tanya.larkina2015@yandex.ru (T.A.L.); ab_poultry@mail.ru (A.B.V.)

**Keywords:** genome-wide association study, chickens, polymorphism, single nucleotide, pigmentation, melanin, feathers, Columbian plumage

## Abstract

Plumage coloration in birds is a complex genetic trait involving both direct pigmentation genes and their modifiers. The Columbian pattern, characterized by black feathers restricted to the neck, wing tips, and tail, remains poorly understood at the genetic level. This study aimed to conduct a genome-wide association study of the Columbian pattern across 29 chicken breeds of diverse origins. Blood samples were collected from breeds with (*n* = 11) and without (*n* = 18) the Columbian pattern. Genotyping was performed using the Illumina Chicken 60K SNP chip, and GWAS was conducted using EMMAX with Bonferroni correction. A total of 10 significant and nine suggestive SNPs on chromosomes GGA1, 2, 5, 7, 10, and 11 were identified. Eight significant SNPs mapped to a ~0.7 Mb region on GGA11 containing *MC1R* and multiple functionally diverse genes involved in melanocyte adhesion (*CDH1*), signaling (*WWP2*, *NFAT5*), transcription (*ZFHX3*, *TCF25*), protein degradation (*PSMD7*, *ATXN1L*), and vesicular trafficking (*AP1G1*). On GGA2, a significant SNP within a lncRNA gene was located in a QTL for yellow plumage, with positional candidates (*ROCK1*, *GREB1L*, *CABLES1*, *TMEM241*, *ANKRD29*) suggesting links to pheomelanin deposition. A suggestive locus near *DLK1* on GGA5 was also identified. This study refines the genetic architecture of the Columbian pattern, implicating a core region on GGA11 modulating melanocyte function and a distinct locus on GGA2 involved in pheomelanin synthesis.

## Simple Summary

Avian plumage exhibits remarkable diversity in coloration and patterning, yet the genetic basis of many specific patterns remains unresolved. One such example is the Columbian plumage pattern, characterized by black pigmentation restricted to the neck, wing tips, and tail. The present study aimed to identify the genetic loci underlying this distinctive pattern. Blood samples were collected from 29 chicken breeds representing both Columbian and non-Columbian phenotypes. Genomic DNA was analyzed to detect chromosomal regions associated with the Columbian pattern. The most strongly associated region was identified on chromosome 11, located in close proximity to the well-known pigmentation gene *MC1R*. This genomic region also contains other candidate genes potentially involved in melanocyte function. The second significant region was detected on chromosome 2, which may influence the deposition of the yellow-red pigment pheomelanin. Our findings indicate that the Columbian pattern emerges from the interaction of multiple genetic factors. These results advance our understanding of the genetic regulation of avian plumage patterning.

## 1. Introduction

The growing interest among biologists in the genetic basis of bird plumage coloration stems from its relevance to sexual dimorphism and the establishment and maintenance of various population polymorphisms [1]. Moreover, uniformity of appearance within a breed is a key trait in poultry [2]. Therefore, identifying genes responsible for plumage color is of particular importance, since they can serve both as breed-specific genetic markers and as breeding trait markers [3].

Plumage coloration exhibits a wide range of variants, necessitating the study of each and the genetic interactions underlying it. This is complicated by the polygenic nature of the trait, which involves multiple interacting genes. Some genes affect pigment distribution across the body, while others influence its zonal patterning within individual feathers, giving rise to patterns such as barring, spotting, or fringing [4,5]. Despite progress, the molecular processes that generate complex patterns (e.g., stripes and spots) and how their variation leads to different phenotypes remain poorly understood [6].

Birds are among the most colorful vertebrates, with their feathers containing various pigments, including melanins, porphyrins, polyenes, and carotenoids [7]. Studies have confirmed that plumage color depends on melanin content produced by melanocytes in feather follicles [8]. Consequently, feathers and feather follicles are ideal tissues for studying the genetic mechanism of color variation. As derivatives of chicken skin, feather follicles are capable of self-renewal, and their proliferation and differentiation lead to feather formation [9,10].

In vertebrates, pigment cells are derived from the neural crest, a transient embryonic cell population that gives rise to a wide range of cell types, including neurons, bone, glia, and cartilage [6]. In birds and mammals, unpigmented precursor cells, melanoblasts, migrate from the neural crest into the epidermis and developing feather and hair follicles [11,12]. Melanogenesis occurs in melanosomes, granule-like organelles within melanocytes that differentiate from melanoblasts [6,13]. Melanocytes, in turn, produce two types of pigments from the amino acid tyrosine: pheomelanin (yellow-red pigment) and eumelanin (black-brown) [14]. These pigments are synthesized and stored within melanosomes before being transferred to keratinocytes for deposition in developing feathers or hair [15,16].

The regulation of melanogenesis after melanocyte migration into follicles is another important step. Plumage differences can arise from mutations in relevant genes or changes in associated molecules such as transcription factors, receptors, structural proteins, enzymes, and growth factors [17]. For example, high expression of *TRP1* and *TRP2/DCT* genes correlates with dark pigmentation in chickens, ducks, quail, pigeons, and geese [18,19].

Although many loci are involved in melanogenesis, one of the major ones is the *E*-locus, which encodes the melanocortin receptor 1 (*MC1R*) and affects the ratio of pheomelanin to eumelanin [20,21]. *MC1R* activation increases eumelanin synthesis, leading to black/brown pigmentation, whereas its inactivation increases pheomelanin, resulting in yellow/red pigmentation [22]. In addition to the *E*-locus, melanin biosynthesis depends on the *C*-locus, which encodes tyrosinase (*TYR*), a key enzyme in melanin production [23]. Proper tyrosinase function is essential; its impairment blocks the melanin synthesis pathway, resulting in an albino phenotype [15]. Low *TYR* expression favors pheomelanin synthesis, while high expression, often stimulated by α-melanocyte-stimulating hormone (α-MSH), promotes eumelanin synthesis [24]. The expression of *TYR* is also influenced by genes such as *SOX10* and *MLPH* [25,26].

Several mutations give rise to three different alleles at the *C*-locus, including a recessive white mutation caused by the insertion of the avian leukemia virus (ev-1) sequence at the end of intron 4 of the *TYR* gene. This mutation inhibits transcription of exon 5, which is crucial for proper melanosomal enzyme location [27].

Another important locus is the *I*-locus, which encodes the pre-melanosomal protein 17 gene (*PMEL17*) on chromosome 33 (GGA33). This protein is required for normal eumelanosome development. The locus has four alleles: *I* (dominant white), *I^S^* (smoky), *I^D^* (grayish brown), and *i* (the wild-type allele, which produces a variety of pigmented plumage) [28].

A wide range of genes involved in melanogenesis has been studied, including *MC1R*, *SLC24A5*, *KITLG*, *PMEL17*, *EDN3*, *SOX10*, *CDKN2A*, and others [28,29,30]. Notably, despite the diversity of genes involved, the final feather patterns—such as transverse striation, stripes, or spots—are primarily controlled by the spatial and temporal distribution of melanin production [31]. One such pattern is the Columbian plumage pattern, first described in 1965. It is characterized by a specific zonal distribution of black pigment on the tail, neck, and wing feathers [32]. Initially thought to be caused by an allele at the *E*-locus, it was later experimentally shown that the responsible gene was not an *E*-locus allele [33]. Therefore, it was designated *Co*. Despite this early genetic mapping, the molecular basis of the Columbian pattern has remained elusive. Classical linkage analysis, while successfully assigning the *Co* locus, is limited by the restricted genetic diversity and recombination events within single pedigrees, often resulting in large candidate intervals [34]. Moreover, the pattern’s dependence on genetic background and its potential polygenic nature, involving interactions between *Co* locus with modifier genes, complicates its dissection using traditional approaches [35].

Nevertheless, the molecular mechanism and genetic basis of this phenotype remain poorly understood. To date, several studies referring to “sub-Columbian” pattern have been published [36,37]. Also, a combined transcriptome and proteome analysis for potential genes regulating Columbian pattern in Yufen I chicken breed was performed [38]; however, the genetic architecture of this trait remains poorly understood.

Hence, the aim of this study was to conduct a genome-wide association study (GWAS) of Columbian plumage pattern across 29 breeds of diverse genetic and geographical-historical origins. By leveraging the deep historical recombination present in a multi-breed cohort, this approach allows for high-resolution mapping of trait-associated loci and the identification of variants that are consistently associated with the phenotype across diverse genetic backgrounds.

## 2. Materials and Methods

### 2.1. Animal Selection

Animal samples were obtained from the Center of Collective Use (CCU) “Genetic collection of rare and endangered chicken breeds” (RRIFAGB, Saint-Petersburg, Russia). A total of 564 blood samples from 29 breeds were used for the study, including Aurora Blue (Au, *n* = 20), Australorp Black Speckled (ABS, *n* = 20), Australorp Black (AB, *n* = 9), Amrock (Ar, *n* = 20), Bantam Mille Fleur (BMF, *n* = 20), Brahma Buff (*n* = 20), Brahma light (BL, *n* = 20), Hamburg Silver Spangled Dwarf (HSSD, *n* = 20), Poland White-Crested Black (PWB, *n* = 18), Naked Neck (NN, *n* = 20), Zagorsk Salmon (ZS, *n* = 19), Leghorn Light Brown (Italian Partridge) (LLB, *n* = 19), Cochin Blue (CB, *n* = 18), Cochin Dwarf (CD, *n* = 20), Leningrad Golden-Gray (LGG, *n* = 20), Leningrad Mille Fleur (LMF, *n* = 21), Red White-tailed Dwarf (RWD, *n* = 18), Minorca (M, *n* = 19), New Hampshire (NH, *n* = 19), Pantsirevka Black (PB, *n* = 17), Pervomay (Pm, *n* = 28), Plymouth Rock Barred (PRB, *n* = 19), Poltava Clay (PC, *n* = 17), Pushkin (Pu, *n* = 20), Rhode Island Red (RIR, *n* = 24), Sussex (S, *n* = 25), Tsarskoye Selo (TS, *n* = 20), Faverolle (F, *n* = 20) and Czech Golden (CG, *n* = 16).

All individuals used in this study were females (hens). The 29 breeds were selected based on the availability in the genetic collection with sufficient sample sizes and high-quality DNA.

### 2.2. Phenotypic Data

**Figure 1:**
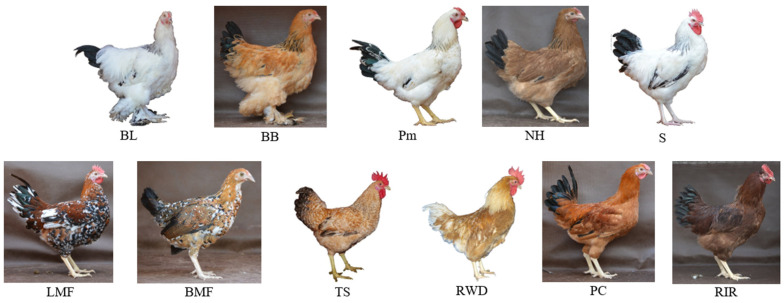
Breeds with Columbian pattern (group 1). BL—Brahma Light, BB—Brahma Buff, Pm—Pervomay, NH—New-Hampshire, S—Sussex, LMF—Leningrad Mille Fleur, BMF—Bantam Mille Fleur, TS—Tsarskoye Selo, RWD—Red White-tailed Dwarf, PC—Poltava Clay, RIR—Rhode Island Red. High-resolution individual photographs of these breeds are provided in Figures S8–S18.

The selection of breeds for the Columbian pattern group was based on their genotype and phenotypic manifestation (Table 1). A total of 11 breeds with Columbian pattern, including Brahma Light, Brahma Buff, Leningrad Mille Fleur, New Hampshire, Poltava Clay, Pervomay, Bantam Mille Fleur, Rhode Island Red, Sussex, Red White-tailed Dwarf, and Tsarskoye Selo, were classified in the first group (Figure 1). High-resolution individual photographs of these breeds are provided in Figures S8–S18. All remaining breeds were assigned to the second group. Notably, the Columbian pattern was fixed in all 11 breeds (100% occurrence), with no segregation of the trait observed over more than 50 generations of purebred selection.

### 2.3. DNA Extraction

Blood samples for DNA extraction were collected at the age of 52 weeks. DNA extraction was carried out by the phenol-chloroform method following standard protocols [39]. The purity and concentration of DNA samples were determined by spectrophotometry on a NanoDrop 2000c (Thermofisher Scientific Inc., Waltham, MA, USA).

### 2.4. Genotyping Data

The Illumina Chicken 60K SNP iSelect BeadChip (Illumina Inc., San Diego, CA, USA) with a coverage density of 57,636 SNPs was used for whole-genome genotyping. Based on the quality control results of genomic reads, samples with at least 95% quality were selected for further analysis. All samples were processed in a single batch to eliminate batch effects. Editing of biochip data to create adaptive extension files (.ped, .map, .fam, .bed, .bim) was performed using PLINK 1.9 software [40] with minor allele frequency (MAF) > 0.05, Hardy–Weinberg equilibrium (HWE) > 0.0001, and missing call rate (geno) > 0.02. After quality control, 46,978 SNPs were selected for the discovery GWAS. The number of SNPs after filtering for each of the subsequent 11 leave-one-out analyses is presented in Supplementary Tables S1–S11.

### 2.5. Population Stratification and Genomic Inflation Control

To quantify and control for population stratification, we calculated the genomic inflation factor (λGC) as the ratio of the median observed χ^2^ statistic to the expected median under the null hypothesis (λGC = median(χ^2^_obs)/χ^2^_0.5, df = 1). We systematically assessed λGC by incrementally including principal components (PCs) as covariates in the linear regression model. The optimal number of PCs was determined by identifying the point at which λGC reached a plateau, balancing the correction for stratification against the potential loss of statistical power. For the λGC and PCA analyses, SNPs were further filtered to remove variants with minor allele frequency (MAF) = 0 or those that failed PCA-specific quality control, resulting in 46,225 SNPs. The discovery GWAS was performed on the full set of 46,978 SNPs passing standard QC.

### 2.6. Genome-Wide Association Study Analysis

To investigate the genetic architecture of Columbian plumage pattern, we conducted a series of GWASs. First, a discovery GWAS was conducted including all 11 breeds with Columbian phenotype. To ensure that associations were not driven by a single breed or population structure, we performed a leave-one-out sensitivity analysis (LOO), i.e., iteratively repeating GWAS, each time excluding one Columbian breed at a time. For each SNP that reached Bonferroni significance (*p* < 1.11 × 10^−6^) in at least one LOO iteration, we recorded the number of iterations in which the SNP remained significant. Replication was classified as full (11/11 iterations), high (≥75%), moderate (≥50%), low (<50%), or none. The complete results of all LOO runs, including the replication status of each SNP, are presented in Supplementary Tables S1–S11 and summarized in Supplementary Table S12. Each GWAS analysis was performed using EMMAX statistical software v. 20120210 [41]. For this purpose, the kinship matrix “identity by state” was generated in EMMAX. The influence of SNPs on the trait was calculated according to the model:

Y=Xb+u+e,

where *Y* is a vector of phenotypes; *b* is an SNP effect; *X* is a design matrix of SNP genotypes; *u* is a vector of additive genetic effects assumed to be normally distributed with the mean equal to 0 and (co)variance *σ2aG*, with *σ2a* being the additive genetic variance and *G* being the genomic relationship matrix; and *e* is a vector of random residual effects.

The significant level for each GWAS run was established using the Bonferroni correction, which allows false positive results to be excluded. Significant and suggestive levels for the discovery GWAS were set as 1.11 × 10^−6^ (0.05/44,978) and 2.22 × 10^−5^ (1.00/44,978), respectively. The significance levels for all of the 11 subsequent leave-one-out analyses are presented in Supplementary Tables S1–S11. Population structure was assessed by principal component analysis (PCA) based on genome-wide SNP data. Visualization of the PCA results was performed using the ggplot2 package v.4.0.3 [42] in the R (v.4.3.2) programming environment. Manhattan and quantile–quantile (Q-Q) plots were generated using the qqman v.0.1.9 [43] and ggplot2 v.4.0.3 packages in the R (v.4.3.2) programming environment.

### 2.7. Linkage Disequilibrium Decay Analysis

To empirically evaluate linkage disequilibrium (LD) decay in our dataset, we calculated the pairwise correlation coefficient *r*^2^ between randomly selected intrachromosomal SNP pairs (within 2 Mb) using Pearson’s correlation of genotype dosages. LD decay was assessed within 100 Kb physical intervals using the R package gaston v.1.6. The half-decay distance (LD½), defined as the physical distance at which the mean *r*^2^ drops to 50% of its maximum, was estimated for the pooled sample (Figure S3) and for each breed with ≥10 samples (*n* = 28 breeds; Figure S4; Table S13).

### 2.8. Annotation of Candidate Genes

Overlapping genes or genes close to the genomic region of candidate SNPs (located within 0.5 Mb of the SNP) were annotated based on the Chicken (Red Jungle Fowl) (Gallus Gallus, GGA) GRCg6a genome assembly. A window size of 0.5 Mb was chosen for annotation. This window size was chosen based on our empirical linkage disequilibrium (LD) decay analysis, which revealed a median breed-specific LD½ of 614 Kb (range: 286–1640 Kb; Figure S4; Table S13), with 68% of breeds (19/28) exhibiting LD½ exceeding 500 Kb. These estimates are consistent with published LD decay ranges for chicken populations (0.5–1 Mb in commercial breeds and 0.1–0.5 Mb in indigenous populations [44,45]). The 0.5 Mb threshold thus represents a conservative distance that captures most physically linked genes and regulatory elements while minimizing spurious associations. SNP information for the corresponding genes was obtained using Ensembl genomic browser.

## 3. Results

**Figure 2:**
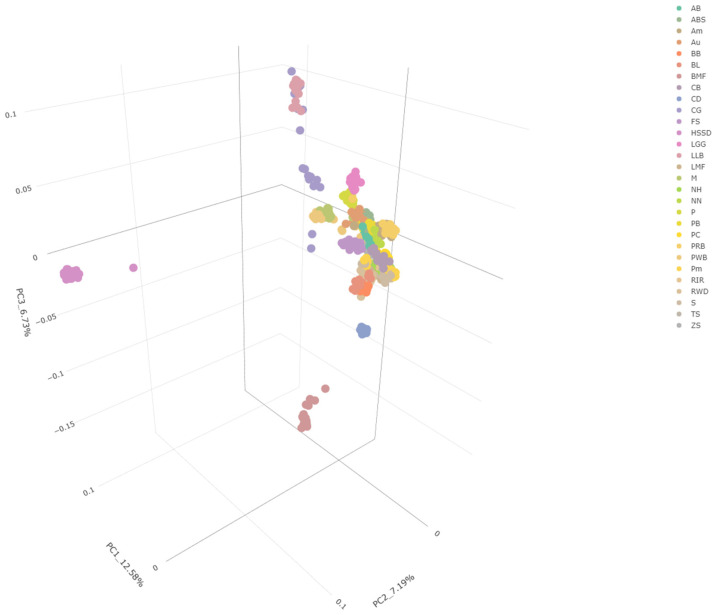
PCA of 564 individuals from 29 chicken breeds. Projection of individuals onto the first three principal components (PC1, PC2, and PC3) is shown.

To visualize the genetic relationships and evaluate potential population stratification, we conducted a PCA on the genome-wide SNP data from all 564 female individuals across 29 breeds. The first three principal components (PC1, PC2, and PC3) explained 12.58%, 7.19%, and 6.73% of the total genetic variance, respectively (Figure 2). The analysis revealed a genetically diverse panel, with breeds segregating largely according to their known geographical origins and breed histories. Columbian pattern representatives were distributed across all three PCs rather than forming a single cluster, indicating that the Columbian phenotype is not simply a reflection of close genetic relatedness among these breeds. This distribution confirms that our case and control groups are well-matched in terms of genetic diversity and supports the robustness of subsequent GWAS findings, as the association signals are unlikely to be driven solely by population stratification.

To assess residual population stratification, we calculated the genomic inflation factor (λGC) for models with varying numbers of principal components (PCs) included as covariates. The λGC decreased from 14.91 in the uncorrected model to a stable plateau of approximately 1.57 when 30 PCs were included (Table 2; Figure S1). Further inclusion of up to 50 PCs did not substantially reduce λGC (range: 1.54–1.57), indicating adequate control of population structure. The Q-Q plot corresponding to the 30-PC model (Figure S2) shows deviation only among the most significant SNPs, consistent with genuine associations rather than a systematic inflation.

**Figure 3:**
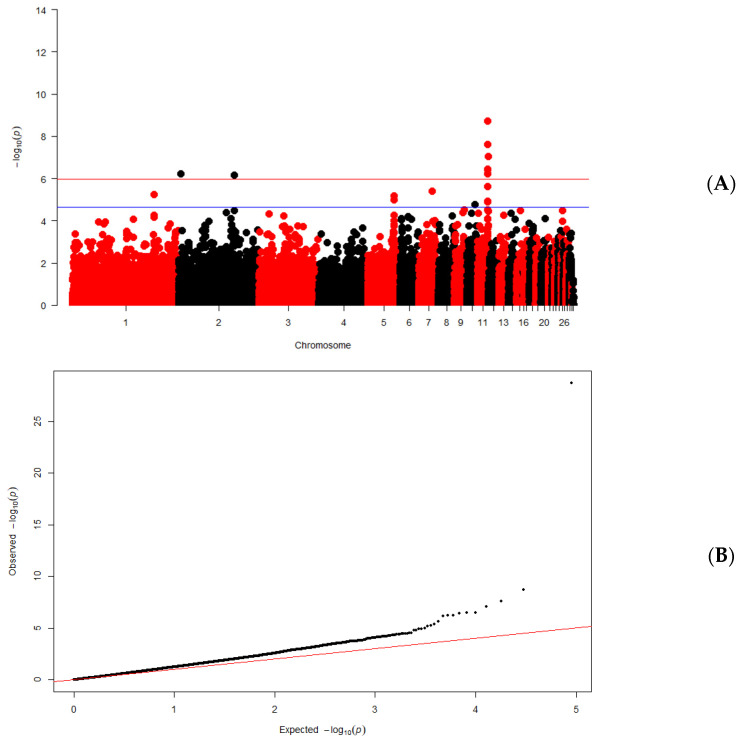
Manhattan (**A**) and Q-Q (**B**) plots for the Columbian plumage pattern GWAS including all 11 breeds. The red and blue horizontal lines in (**A**) indicate the genome-wide significant (*p* < 1.11 × 10^−6^) and suggestive (*p* < 2.22 × 10^−5^) thresholds, respectively. In the Q-Q plot (**B**), the red line indicates the expected distribution under the null hypothesis, and black points indicate the observed *p*-values. Deviation of black points from the red line at high expected *p*-values suggests genuine associations rather than systematic inflation.

A total of 19 SNPs exceeded the predefined thresholds: 10 SNPs were genome-wide significant, and 9 were suggestive (Figure 3 and Figure S5). Of the 10 significant SNPs, two mapped to GGA2 and the remaining 8 to GGA11 (Table 3). Suggestive SNPs were mainly located on chromosomes 1 (1 SNP), 5 (2 SNPs), 7 (1 SNP), 10 (2 SNPs), and 11 (3 SNPs) (Table 4).

To evaluate the robustness of our findings and assess the influence of individual breeds, we performed a leave-one-out (LOO) sensitivity analysis, iteratively repeating the GWAS while excluding one of the 11 Columbian breeds at a time. Across the 11 LOO iterations, 49 unique SNPs reached Bonferroni significance (*p* < 1.11 × 10^−6^) in at least one analysis. Of these, four SNPs were fully replicated in all 11 iterations, all mapping to a ~0.7 Mb region on GGA11 (18.8–19.5 Mb): rs15626912, rs13775909, rs14029392, and rs315879267 (Table 5). These SNPs represent high-confidence, breed-independent associations. Two additional SNPs showed high replication (≥75% of iterations): rs14130009 (GGA2) and rs16058057 (GGA2). The majority of SNPs (57%) were significant only in specific LOO subsets, indicating that their signals were influenced by the exclusion of particular breeds. The breeds whose exclusion most frequently led to loss of significance included Rhode Island Red (RIR) and Pervomay (Pm).

A summary of replication counts for all 49 SNPs is provided in Supplementary Table S12. The distribution of SNPs across replication categories (Figure S6) and the histogram of replication counts (Figure S7) further illustrate the robustness of the GGA11-associated SNPs.

LD decay analysis revealed rapid decay in the pooled sample (LD½ = 169 Kb; Figure S3), reflecting the genetic heterogeneity of the multi-breed panel. Within-breed LD decay was significantly slower, with a median LD½ of 614 Kb (range: 286–1640 Kb; Figure S4; Table S13). In 68% of breeds (19/28), LD½ exceeded 500 Kb, supporting the use of a 0.5 Mb candidate gene window.

While we did not formally estimate the proportion of phenotypic variance explained by individual SNPs, the high replication rate of the GGA11 SNPs across all 11 LOO iterations (4/4 SNPs fully replicated) and their location within a single LD block containing the well-known pigmentation gene *MC1R* suggest that this region constitutes a major effect locus for the Columbian pattern. Formal estimation of SNP-based heritability using GCTA will be the focus of future studies.

## 4. Discussion

Eight of the 11 significant SNPs were located within a specific 0.7 Mb region on GGA11 (18.8–19.5 Mb). LD block analysis revealed that these significant SNPs reside within a single extended haplotype block, suggesting that the association signal is likely driven by a single causal variant rather than independent contributions from multiple genes. The most plausible candidate is the well-established pigmentation gene *MC1R*, which has been repeatedly associated with plumage coloration in chicken [46,47,48,49,50] and affects pigmentation of both skin [51,52,53] and feathers [21,54,55], as well as feather patterning [56]. While historically the *Co* locus was not considered an allele of the *E*-locus, our findings demonstrate that the causative variant(s) for the Columbian pattern are in strong LD with *MC1R*. This could be explained by two non-mutually exclusive scenarios: (1) *MC1R* acts as a master regulator of a pigmentation network, where the Columbian phenotype arises from complex interactions between the *MC1R* locus and other modifiers; or (2) the true causal variant lies in a regulatory element affecting *MC1R* expression. The involvement of other genes in the LD block may reflect their roles in the same regulatory network or be a consequence of LD with the primary signal. Nevertheless, given the strong LD, the functional relevance of individual genes within this region should be interpreted with caution. The 0.7 Mb region can be conventionally divided into 2 separate sub-regions. The first sub-region contained seven SNPs (18.8–19.1 Mb) located within protein-coding genes (*GAS8*, *CDH1*, *WWP2*, *PSMD7*) and long non-coding RNA genes (lncRNAs). One of the SNPs, rs15626912, is located within the *GAS8* gene, which is a member of *ZFP276-MC1R-GAS8* linkage group common to humans, chickens, dogs and chimpanzees [57]. Although *GAS8* is known to be involved in the proper functioning of motile cilia [58], its association with plumage color is more likely attributable to its physical linkage to *MC1R*. Several other pigmentation-associated genes are in close proximity to rs15626912, including *DEF8*, *TCF25*, and *CDK10* genes. *DEF8* has been involved in pigmentation pathways in a GWAS of actinic keratosis [59]. *TCF25*, a transcription factor involved in embryonic development [60], has recently been associated with mottled plumage in Liangshan Yanying chicken breed [46] and with skin pigmentation in sheep and ducks [61,62]. *CDK10*, a Ser/Thr protein kinase, shows expression associations with hair color, melanoma, and basal cell carcinoma in humans [63]. The presence of multiple pigmentation-associated genes within this region suggests that Columbian pattern may be influenced by a complex interaction of genes, involved in melanocyte development, survival, and function.

Another SNP (rs3137644) is located within *CDH1*, a gene that encodes a calcium-dependent cell adhesion molecule. In human vitiligo, a specific *CDH1* mutation leads to impaired melanocyte adhesion and localized pigment loss [64]. *CDH1* has also been associated with chicken plumage coloration, particularly with black plumage in Yuexi Frizzled breed [65] and with golden/silver feathering in the Wenchang breed [66]. These data suggest that *CDH1* could act as a modifier gene affecting pigment distribution via melanocyte attachment in feather follicles. This is particularly relevant for the Columbian pattern with its sharp boundaries between pigmented and non-pigmented feather parts. This hypothesis is supported by transcriptomic evidence implicating the calcium signaling pathway in Columbian pattern development [38].

Another SNP (rs15627346) from the first sub-region is localized within *WWP2*, which encodes an E3 ubiquitin ligase known to target *PTEN* for proteasomal degradation. A study by Cao et al. (2013) identified a functional link between *WWP2*, *PTEN*, and *MC1R* genes [67]. *PTEN* is a key regulator of the PI3K/AKT signaling pathway important for cell proliferation, migration and survival. The MC1R receptor binds to PTEN, protecting it from degradation by WWP2. Based on these data, we propose several hypotheses. First, this particular *WWP2* variant might affect the efficiency of *PTEN* binding and degradation in specific areas, leading to zonal pigment loss. Second, *WWP2* could act as a modifier gene, where *MC1R* signaling differs between pigmented and non-pigmented regions: *MC1R* protects *PTEN* in pigmented areas, while *WWP2* degrades *PTEN* in non-pigmented ones, disrupting melanocyte survival or function. Finally, given the location of the *WWP2* variant in close proximity to *MC1R*, this SNP signal might reflect LD with the causal variant near *MC1R*.

Another SNP within the first sub-region on GGA11 is localized within the *PSMD7* gene, which participates in the degradation of ubiquitinated proteins—a pathway also involving *WWP2*. Taken together, these data may indicate a specific role for proteasomal regulation of pigmentation-related factors.

Beyond protein-coding genes, three significant SNPs are localized within intronic regions of lncRNAs. Although the functional roles of these lncRNAs remain unknown, their presence within this associated interval suggests they may contribute to the regulation of nearby pigmentation-related genes. Among the protein-coding genes, *NQO1* and *NFAT5* warrant special attention. *NQO1* encodes an oxidoreductase identified as a positive regulator of melanogenesis in human and zebrafish [68]. *NQO1* inhibition significantly reduces pigmentation, while its overexpression induces melanogenesis by increasing *TYR* levels through suppression of its degradation [69]. Although the role of *NQO1* in avian species pigmentation has not been studied, its role as a positive melanogenesis regulator makes it a strong candidate for plumage color patterning. The identified SNPs near *NQO1* might affect its activity, contributing to the specific pigment loss of the Columbian pattern. *NFAT5* encodes a transcription factor that regulates cellular responses to osmotic stress [70]. Although no direct link between *NFAT5* and pigmentation has been found, several indirect connections are worth mentioning: *NFAT5* is active in retinal pigment epithelium cells, where osmotic homeostasis is vital for proper cell functioning, and cellular stress responses are known to influence melanocyte biology and melanogenesis [71]. This suggests that *NFAT5* may influence melanocyte adaptation to the local microenvironment in different feather zones, potentially contributing to the formation of distinct boundaries between pigmented and non-pigmented areas. However, this hypothesis requires experimental validation.

The second sub-region contained the last significant SNP on GGA11, located within *ZFHX3*—a transcription factor that can act both as an activator and repressor [72]. Along with *CDH1* and *TCF25*, *ZFHX3* has been recently proposed as a candidate gene associated with plumage coloration in the Yuexi frizzled chicken breed [65]. Furthermore, *ZFHX3* falls within a runs of homozygosity (ROH) region identified in the Korean Yeonsan Ogye chicken breed, a genomic block associated with QTLs for skin and comb coloration that includes several pigmentation-related genes discussed above, namely *TCF25*, *MC1R*, *DEF8*, *GAS8*, *CDH1*, *NFAT5*, *WWP2*, and *PSMD7* [73]. In addition to these, this ROH island also contains *AP1G1* and *ATXN1L*, located in close proximity to the significant SNP within *ZFHX3*. *AP1G1* encodes a subunit of the adaptor protein complex 1 (AP-1), which plays a critical role in intracellular trafficking. This function is particularly important for pigmentation, as the AP-1 complex is essential for the proper transport of melanogenic enzymes (such as tyrosinase) and other melanosomal proteins to melanosomes [74]. Variants affecting *AP1G1* expression might alter the efficiency of this trafficking machinery, contributing to the specific pattern of pigment deposition. *ATXN1L* is thought to be involved in protein–protein interactions and transcriptional regulation [75], potentially through its association with ubiquitination pathways [76]. Its location within this pigmentation-associated ROH island and its potential role in protein stability and degradation pathways lead to its consideration as a modifier gene. In the context of Columbian pattern, *ATXN1L* might influence the stability of key pigment enzymes or transcription factors, which might lead to specific pigmentation output. Taken together, the comparison of independent GWAS and ROH mapping underscores the importance of this particular region on GGA11 for pigmentation of skin and its derivatives in chicken.

The remaining two significant SNPs are located in non-coding regions on GGA2: rs14130009 lies within an intergenic region, and rs317732749 resides within a lncRNA gene. Of particular interest is the rs317732749, which maps to a prominent QTL previously associated with yellow plumage coloration in chickens. This particular QTL contains several protein-coding genes in close proximity to the SNP, including *ROCK1*, *GREB1L*, *CABLES1*, *TMEM241*, and *ANKRD29*, which have been proposed as candidate genes for pigmentation [77]. The biological functions of these genes suggest potential mechanisms by which they could influence pheomelanin deposition in the Columbian pattern. *ROCK1*, a key regulator of the actin cytoskeleton, might modulate dendrite formation in melanocytes, thereby affecting melanosome transport to feather keratinocytes [78]. *GREB1L* acts as a coactivator for retinoic acid receptors; retinoic acid, a derivative of carotenoids, is known to influence both melanocyte differentiation and the type of melanin synthesized, indicating its direct involvement in pigmentation [79]. *CABLES1* interacts with tyrosine kinase signaling pathways, including the KIT receptor, which is fundamental for melanocyte survival and migration [80]. To date, the specific roles of *TMEM241* and *ANKRD29* in pigmentation are not yet characterized; however, their presence within the yellow plumage QTL requires further analysis. Taken together, these data suggest that rs317732749 may serve as a marker for a functionally important haplotype contributing to the Columbian plumage pattern, likely through modulation of pheomelanin deposition.

In addition to the significant SNPs, GWAS identified nine suggestive SNPs on GGA1, 5, 7, 10, and 11 (Table 4). Since the suggestive SNPs on GGA11 fall within the same region as the significant SNPs, they are not discussed further to avoid redundancy. The majority of SNPs, namely on GGA1, 7, and 10, are located within lncRNA genes, the functional relevance of which remains to be explored. Of particular interest is the GGA5, which contains two SNPs: rs14545421, located within a DNA-coding gene with unknown function, and rs13590535, located within a lncRNA gene near *DLK1*—a gene, recently associated with plumage coloration in the Yuexi frizzled chicken breed [65]. Although the specific functions of all of the lncRNAs in melanogenesis are not yet characterized, their presence in a GWAS for plumage color suggests they may harbor regulatory variants influencing the Columbian phenotype.

While our GWAS identifies robust associations between specific SNPs and the Columbian pattern, we acknowledge that association does not equate to causation. Several important limitations should be considered when interpreting these findings.

First, the Columbian phenotype group includes breeds carrying additional pigmentation modifiers, such as *mo*, *I*, and *B* (Table 1). While our strict phenotype definition, multi-breed GWAS design, and LOO sensitivity analysis help mitigate the confounding effects of these modifiers, we cannot completely exclude their influence. The detected signals, particularly on GGA11, are robust across most LOO iterations, suggesting they primarily reflect the Columbian pattern itself rather than a composite of different modifier signals. Nevertheless, future fine-mapping studies in isogenic backgrounds will be required to dissect the individual contributions of these modifiers.

Second, the candidate genes proposed here—including *CDH1*, *WWP2*, *ZFHX3*, and others—require functional validation to confirm their role in pigment patterning. While our findings are supported by biological plausibility and previous associations with pigmentation in other species, direct experimental evidence in chickens is currently lacking. Future studies using CRISPR/Cas9-mediated gene editing in chicken models, RNA interference in melanocyte cultures, or expression analyses across different feather tracts would provide definitive evidence for the molecular mechanisms underlying the Columbian pattern.

Third, the absence of an independent external cohort for replication is a limitation of this study. Future validation of these loci in geographically and genetically distinct chicken populations is required to confirm their generalizability.

Fourth, we recognize that classifying individuals at the breed level can introduce biases in a multi-breed GWAS. To mitigate this, we applied a mixed model (EMMAX) incorporating a kinship matrix and used PCA with 30 PCs to control for population stratification. The substantial reduction in λGC after correction (from 14.91 to 1.57; Table 2) indicates that our approach effectively accounts for breed structure. However, we cannot entirely exclude the possibility that some associations are influenced by breed-specific genetic backgrounds.

Lastly, a limitation of this study is that we did not formally estimate the proportion of phenotypic variance explained by the identified loci. Future studies employing GCTA or similar approaches will be required to quantify the contribution of these loci to the Columbian phenotype.

## 5. Conclusions

The first multi-breed GWAS for Columbian plumage pattern identified a total of 10 significant and nine suggestive SNPs across chromosomes GGA1, 2, 5, 7, 10, and 11. A rigorous correction for population stratification and extensive leave-one-out sensitivity analysis across 11 Columbian breeds confirmed the robustness of the major associations. On GGA11, eight significant SNPs delineate a ~0.7 Mb region containing the well-known pigmentation gene *MC1R* and a cluster of functionally diverse candidates (*CDH1*, *WWP2*, *NFAT5*, *ZFHX3*, *TCF25*, *PSMD7*, *ATXN1L*, *AP1G1*), supported by independent ROH mapping data [73]. LD block analysis revealed that these SNPs reside within a single extended haplotype block, suggesting that the Columbian pattern is likely driven by a primary causal variant in or near *MC1R*, potentially acting through complex interactions with other genes in this regulatory network. Notably, four SNPs in this region—rs15626912, rs13775909, rs14029392, and rs315879267—were fully replicated in all 11 LOO iterations, confirming their status as high-confidence, breed-independent associations. On GGA2, a significant SNP within a lncRNA gene (rs317732749) maps to a major QTL for yellow plumage [77], with positional candidates (*ROCK1*, *GREB1L*, *CABLES1*, *TMEM241*, *ANKRD29*) suggesting mechanisms influencing pheomelanin deposition. Nine suggestive SNPs, including the locus near *DLK1* on GGA5, provide a foundation for future fine-mapping and functional studies.

This study refines the genetic architecture of the Columbian plumage pattern, implicating a core set of genes on GGA11 involved in melanocyte biology and a distinct locus on GGA2 linked to pheomelanin synthesis.

## Figures and Tables

**Table 1:** Columbian pattern breeds: genotype and phenotypic manifestation.

Breed	Genotype **	Phenotypic Manifestation
Bantam Mille Fleur (BMF)	*Co/Co* *s/s* *mo/mo* *(e^b^*, *e^Wh^*, *e^+^)*	Mille fleur plumage is caused by the combined action of “*Co*”, “*mo*” and “*s*” alleles on a background of any of the three recessive “*e*”: *e^b^*, *e^Wh^*, or *e^+^*. “*Co*” restricts melanin to the neck, wing tips, and tail feathers and acts on a recessive *E*-locus background; “*s*” determines the main golden background color; “*mo*” (mottling) in combination with “*Co*” and “*s*”, produces a black transverse stripe and white tip (“pearl”) on each feather, resulting in the mille fleur pattern.
Leningrad Mille Fleur (LMF)		
Brahma Buff (BB) *	*Co/Co* *e^b^/e^b^* *s/s*	“*Co*” restricts melanin production to the neck, wing tips, and tail feathers; “*s*” determines the main golden background color, while “*e^b^*” modifies it to a brownish hue.
Brahma Light (BL) *	*Co/Co* *e^b^/e^b^* *S/S*	“*Co*” restricts melanin production, leaving black plumage only on the neck, wing tips, and tail; “*S*” allele determines the main silver background color.
New-Hampshire (NH) *	*Co/Co* *s/s* *e^Wh^/e^Wh^*	“*Co*” restricts melanin to the neck, wing tips, and tail feathers; “*s*” allele determines the main golden background color. “*Co*” and “*e^Wh^*” interaction modifies the main color to a pale reddish-golden hue.
Pervomay (Pm)	*Co/Co* *S/S*	“*Co*” restricts melanin to the neck, wing tips, and tail feathers; “*S*” determines the main silver background color.
Poltava Clay (PC) *	*Co/Co* *s/s* *e^Wh^/e^Wh^*	“*Co*” restricts melanin production to the neck, wing tips, and tail feathers; “*s*” determines the main golden background color; “*Co*” and “*e^Wh^*” interaction modifies the main color to a reddish-golden hue.
Red White-Tailed Dwarf (RWD) *	*Co/Co* *e^b^/e^b^* *s/s* *I/I*	“*Co*” restricts melanin production to the neck, wing tips, and tail feathers; “*s*” determines the main golden background color, while “*e^b^*” modifies it to a brownish hue; “*I*” restricts melanin production, as a result of which the “Columbian” zones of black plumage (neck, wing tips, and tail feathers) become white.
Rhode Island Red (RIR) *	*Co/Co* *e^b^/e^b^* *s/s*	“*Co*” restricts melanin production to the neck, wing tips, and tail feathers; “*s*” determines the main golden background color, while “*e^b^*” modifies it to a brownish hue.
Sussex (S)	*Co/Co* *S/S*	“*Co*” restricts melanin to the neck, wing tips, and tail feathers; “*S*” determines the main silver background color.
Tsarskoye Selo (TS)	*Co/Co* *e^Wh^/e^Wh^* *s/s* *mo/mo* *B/B*	“*Co*” restricts melanin to the neck, wing tips, and tail feathers. The autosexing white-barred plumage coloration is caused by the combined action of “*Co*”, “*B*” (barring), and the recessive “*mo*” (mottling), providing white “pearls” on feather tips and enhancing white transverse stripes. The “*s*” determines the main golden background color, while the “*e^Wh^*” modifies it to a buff (wheaten) hue.

* When folded, the flight feathers hide the black parts of the Columbian plumage. ** For each breed, the genotype column lists alleles at the *Co*, *s*, *mo*, *E*, *I*, and *B* loci. The phenotypic manifestation column describes the contribution of each locus; the Columbian pattern arises from the combined action of these loci, with *Co* serving as the primary determinant.

**Table 2:** Genomic inflation factor (λGC) and number of significant SNPs after correction for population stratification using increasing numbers of principal components (PCs).

PCs	λGC	Bonferroni Significant SNPs *	FDR Significant SNPs *
0	14.91	9332	26,371
5	9.99	5470	21,927
10	5.47	1598	13,669
20	2.68	127	3303
**30**	**1.57**	**6**	**82**
40	1.55	2	95

* Number of SNPs exceeding the significance threshold after Bonferroni correction (*p* < 1.11 × 10^−6^) or FDR correction (*q* < 0.05). Bold indicates the selected 30 PCs, where λGC plateaued.

**Table 3:** Significant SNPs associated with Columbian plumage pattern.

SNP	GGA *	Position (b.p.)	*p*-Value	Motif	GENE	GENE Nearby
rs14130009	2	3820500	5.87 × 10^−7^	C/A	*intergenic*	
rs317732749	2	102689611	6.92 × 10^−7^	A/C/G	lncRNA	*ROCK1* *GREB1L* *CABLES1* *TMEM241* *ANKRD29*
rs15626912	11	18853914	2.36 × 10^−6^	T/C	*GAS8*	*MC1R* *DEF8* *TCF25* *CDK10*
rs3137644	11	18876832	3.83 × 10^−7^	C/T	*CDH1*	
rs15627346	11	19066964	3.49 × 10^−7^	C/T	*WWP2*	*NQO1* *NFAT5*
rs315288757	11	19079133	3.49 × 10^−7^	C/T	*PSMD7*	
rs14968579	11	19150630	5.90 × 10^−7^	C/T	lncRNA	
rs13775909	11	19159311	1.84 × 10^−9^	G/A	lncRNA	
rs14029392	11	19173660	2.42 × 10^−8^	T/C	lncRNA	
rs315879267	11	19521295	8.72 × 10^−8^	C/T	*ZFHX3*	*NFAT5* *ATXN1L* *AP1G1*

* GGA—chromosome number for Gallus Gallus Domesticus; *p*-values are from the discovery GWAS including all 11 Columbian breeds; motif indicates the reference/alternative alleles.

**Table 4:** Suggestive SNPs associated with Columbian plumage pattern.

SNP	GGA *	Position (b.p.)	*p*-Value	Motif	GENE	GENE Nearby
rs13957213	1	152154067	5.70 × 10^−6^	G/A	lncRNA	
rs14545421	5	48752720	1.00 × 10^−5^	C/T	DNA-coding	
rs13590535	5	49435813	6.42 × 10^−6^	T/C	lncRNA	*DLK1*
rs14618569	7	24848594	3.95 × 10^−6^	C/T	lncRNA lncRNA	
rs736654754	10	15224421	1.74 × 10^−5^	G/T	lncRNA lncRNA	
rs741363378	10	15224422	1.74 × 10^−5^	A/G	lncRNA lncRNA	
rs15627312	11	19028643	2.35 × 10^−6^	T/C	*NFAT5*	*GAS8* *MC1R* *DEF8* *TCF25* *PSMD7* *WWP2* *NQO1* *NOB1* *ZFHX3*
rs13775890	11	19103008	1.20 × 10^−5^	A/G	lncRNA	
rs14029401	11	19178856	1.28 × 10^−5^	G/A	upstream gene variant	

* GGA—chromosome number for Gallus Gallus Domesticus; *p*-values are from the discovery GWAS including all 11 Columbian breeds; motif indicates the reference/alternative alleles.

**Table 5:** Leave-one-out replication summary for SNPs with ≥50% replication rate.

SNP	Position (b.p.)	GGA	Min *p*-Value	Max *p*-Value	Replication	Status	Lost When Excluding
rs15626912	18853914	11	3.34 × 10^−35^	3.00 × 10^−27^	11/11 (100%)	Full	None
rs13775909	19159311	11	6.71 × 10^−11^	2.00 × 10^−8^	11/11 (100%)	Full	None
rs14029392	19173660	11	3.17 × 10^−9^	2.57 × 10^−7^	11/11 (100%)	Full	None
rs315879267	19521295	11	1.37 × 10^−8^	9.92 × 10^−7^	11/11 (100%)	Full	None
rs15627346	19066964	11	3.97 × 10^−7^	2.21 × 10^−6^	9/11 (81.8%)	High	Pm, RIR
rs315288757	19079133	11	3.98 × 10^−7^	1.91 × 10^−6^	9/11 (81.8%)	High	Pm, RIR
rs3137644	18876832	11	1.71 × 10^−8^	1.57 × 10^−6^	8/11 (72.7%)	Moderate	Pm, S, TS

## Data Availability

The data presented in this study are included in the article and the Supplementary Materials. The datasets generated and analyzed during the current study are available on request from the corresponding author.

## Supplementary Materials

The following supporting information can be downloaded at: https://www.mdpi.com/article/10.3390/ani16142153/s1, Table S1: GWAS leave-one-out sensitivity analysis without the Brahma Buff breed; Table S2: GWAS leave-one-out sensitivity analysis without the Brahma Light breed; Table S3: GWAS leave-one-out sensitivity analysis without the Leningrad Mille Fleur breed; Table S4: GWAS leave-one-out sensitivity analysis without the New-Hampshire breed; Table S5: GWAS leave-one-out sensitivity analysis without the Poltava Clay breed; Table S6: GWAS leave-one-out sensitivity analysis without the Pervomay breed; Table S7: GWAS leave-one-out sensitivity analysis without the Rhode Island Red breed; Table S8: GWAS leave-one-out sensitivity analysis without the Bantam Mille Fleur breed; Table S9: GWAS leave-one-out sensitivity analysis without the Red White-Tailed Dwarf breed; Table S10: GWAS leave-one-out sensitivity analysis without the Sussex breed; Table S11: GWAS leave-one-out sensitivity analysis without the Tsarskoye Selo breed; Table S12: Full leave-one-out replication summary for all 49 SNPs; Table S13: Linkage disequilibrium half-decay (LD½) estimates by breed; Supplementary Figures: Figure S1: Genomic inflation factor (λGC) as a function of the number of principal components (PCs) included as covariates in the GWAS model. λGC decreased from 14.91 (uncorrected) to a stable plateau of ~1.57 with 30 PCs; Figure S2: Quantile–quantile (Q-Q) plot for the 30-PC model (λGC = 1.57); Figure S3: Linkage disequilibrium (LD) decay in the pooled sample (LD½ = 169 Kb); Figure S4: Linkage disequilibrium half-decay (LD½) estimates for each breed; Figure S5: Manhattan plot after correction with 30 principal components (λGC = 1.57); Figure S6: Distribution of SNPs across leave-one-out replication categories; Figure S7: Histogram of leave-one-out replication counts for all 49 SNPs; Figures S8–S18: High-resolution photographs of the 11 chicken breeds exhibiting the Columbian plumage pattern. Figure S8—Brahma Light; Figure S9—Brahma Buff; Figure S10—Sussex; Figure S11—New-Hampshire; Figure S12—Pervomay; Figure S13—Leningrad Mille Fleur; Figure S14—Bantam Mille Fleur; Figure S15—Tsarskoye Selo; Figure S16—Red White-tailed Dwarf; Figure S17—Poltava Clay; Figure S18—Rhode Island Red.

## Abbreviations

The following abbreviations are used in this manuscript:

Au	Aurora Blue
ABS	Australorp Black Speckled
AB	Australorp Black
Ar	Amrock
BMF	Bantam Mille Fleur
BB	Brahma Buff
BL	Brahma Light
HSSD	Hamburg Silver Spangled Dwarf
PWB	Poland White-Crested Black
NN	Naked Neck
ZS	Zagorsk Salmon
LLB	Leghorn Light Brown (Italian Partridge)
CB	Cochin Blue
CD	Cochin Dwarf
LGG	Leningrad Golden-Gray
LMF	Leningrad Mille Fleur
RWD	Red White-tailed Dwarf
M	Minorca
NH	New Hampshire
PB	Pantsirevka Black
Pm	Pervomay
PRB	Plymouth Rock Barred
PC	Poltava Clay
Pu	Pushkin
RIR	Rhode Island Red
S	Sussex
TS	Tsarskoye Selo
F	Faverolle
CG	Czech Golden
GWAS	Genome-wide association study
SNP	Single nucleotide polymorphism
QTL	Quantitative trait loci
GGA*X*	Chicken autosome *X*
CCU	Center of Collective Use
MAF	Minor allele frequency
HWE	Hardy–Weinberg equilibrium
LD	Linkage disequilibrium
PCA	Principal component analysis
