# Supplementary material for: Genomic Analysis of the Columbian Plumage Pattern in Various Chicken Breeds
Source: Animals (Basel). 2026 Jul 11;16(14):2153. doi: 10.3390/ani16142153 (PMC13404467; doi:10.3390/ani16142153)
Supplement: Supplementary file 1 [file animals-16-02153-s001.zip › Figure S8-18.pdf]

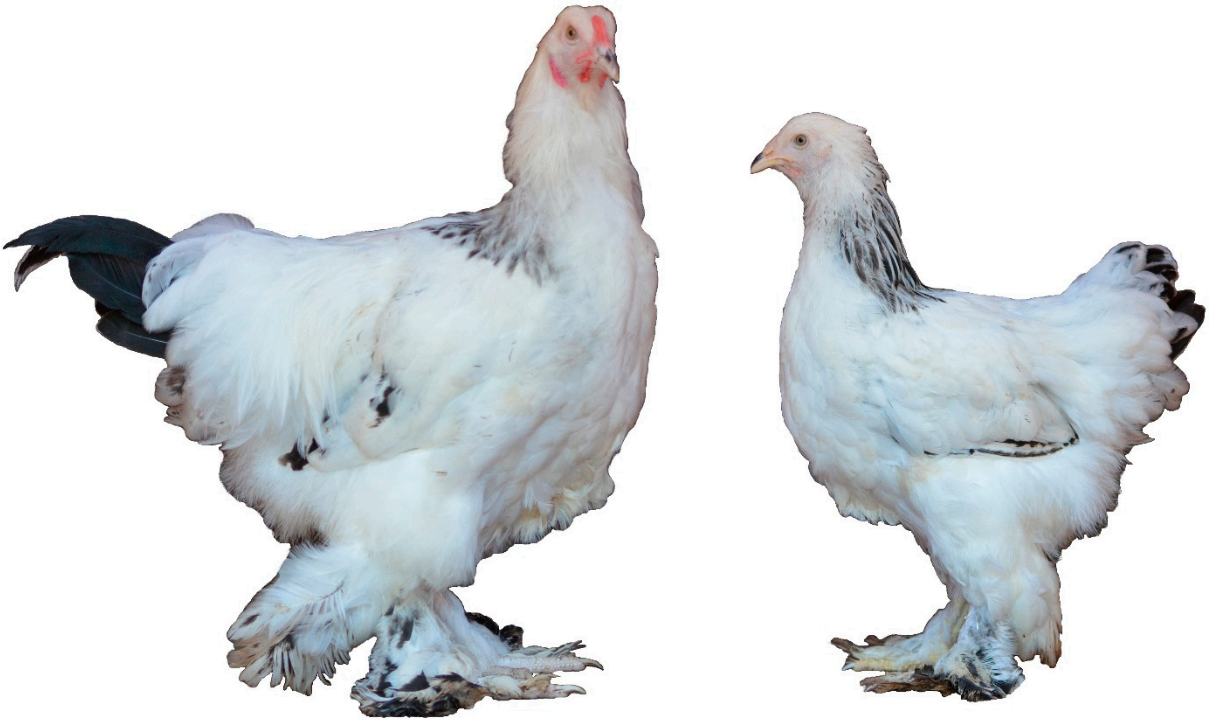

Figure S8. Brahma Light chicken breed

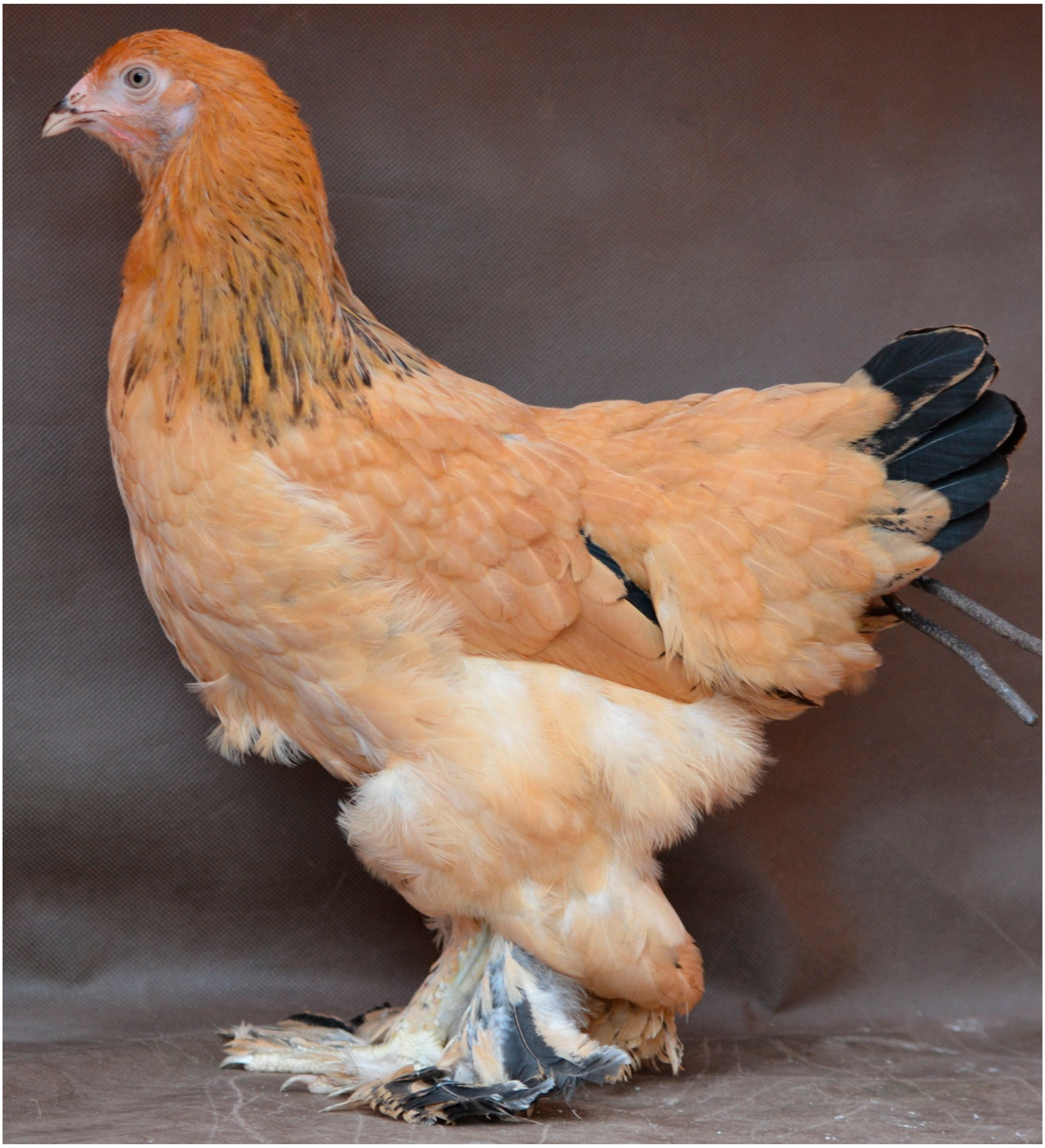

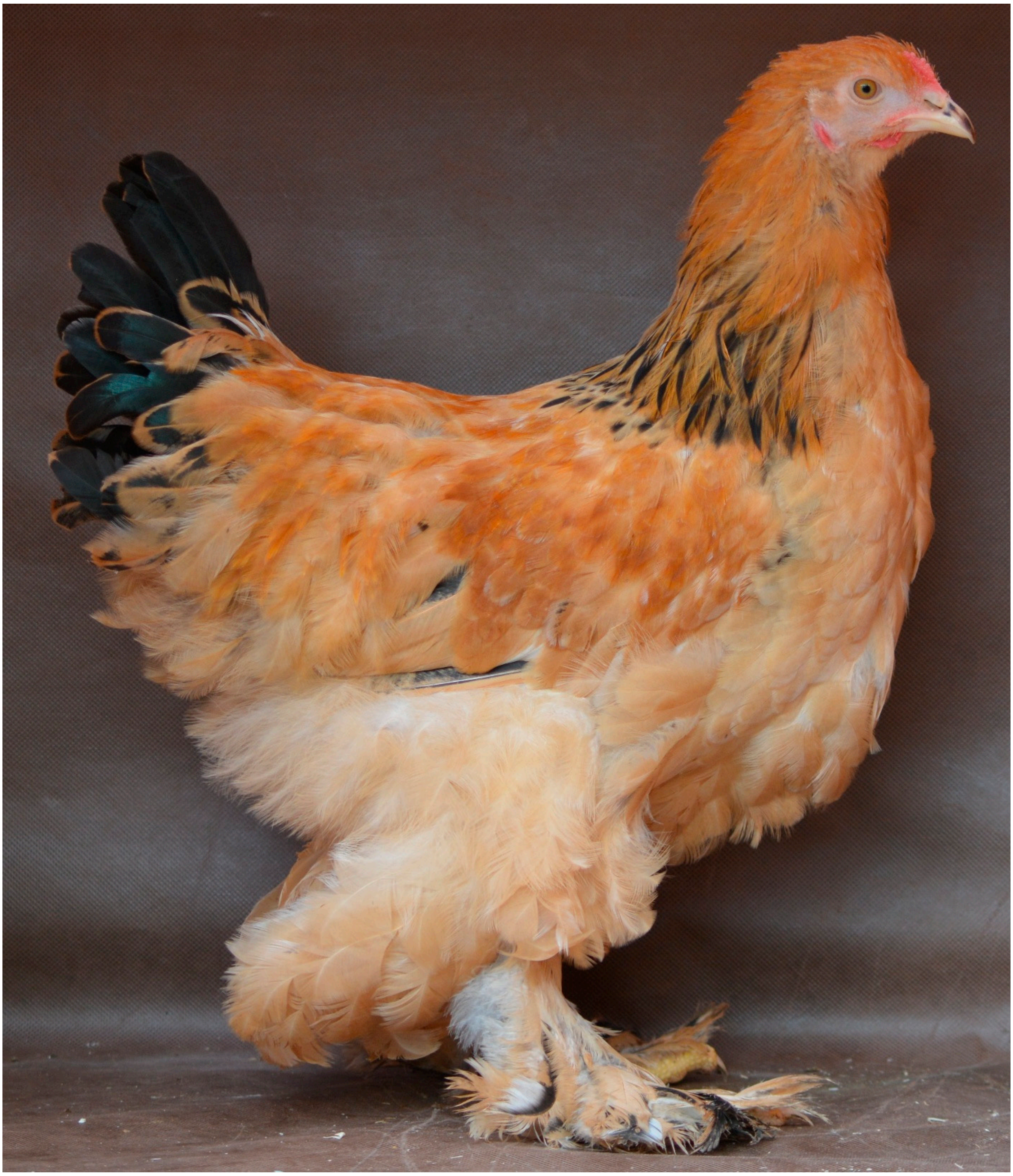
Figure S9. Brahma Buff chicken breed

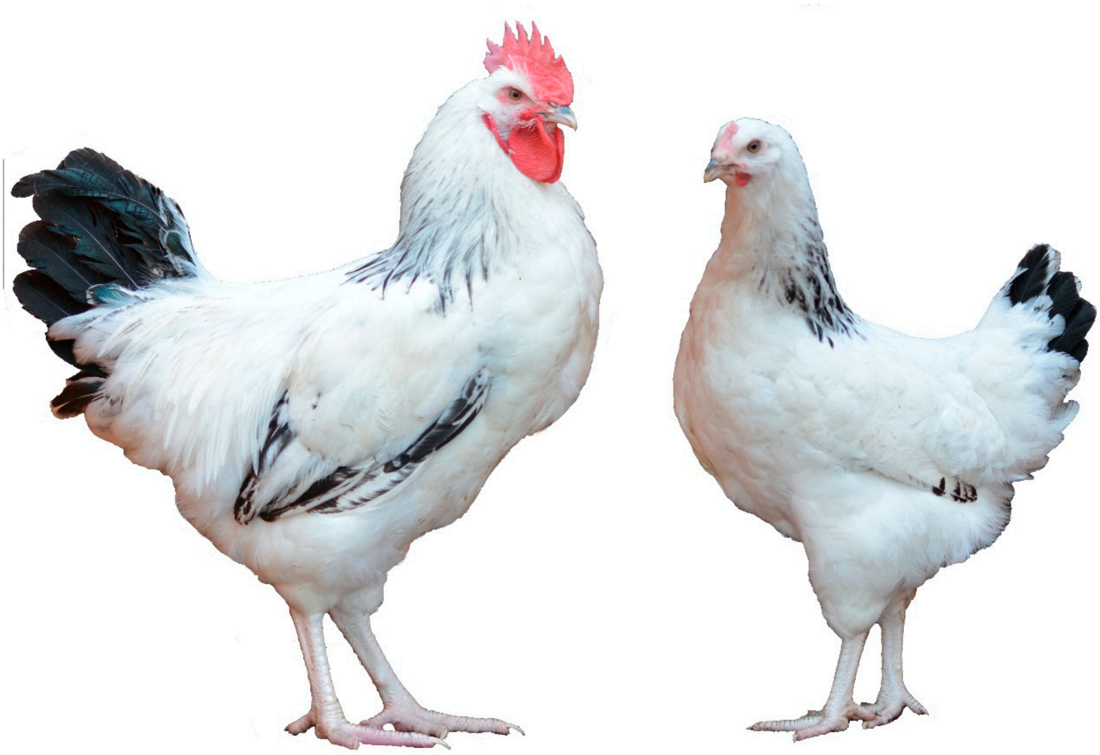

Figure S10. Sussex chicken breed

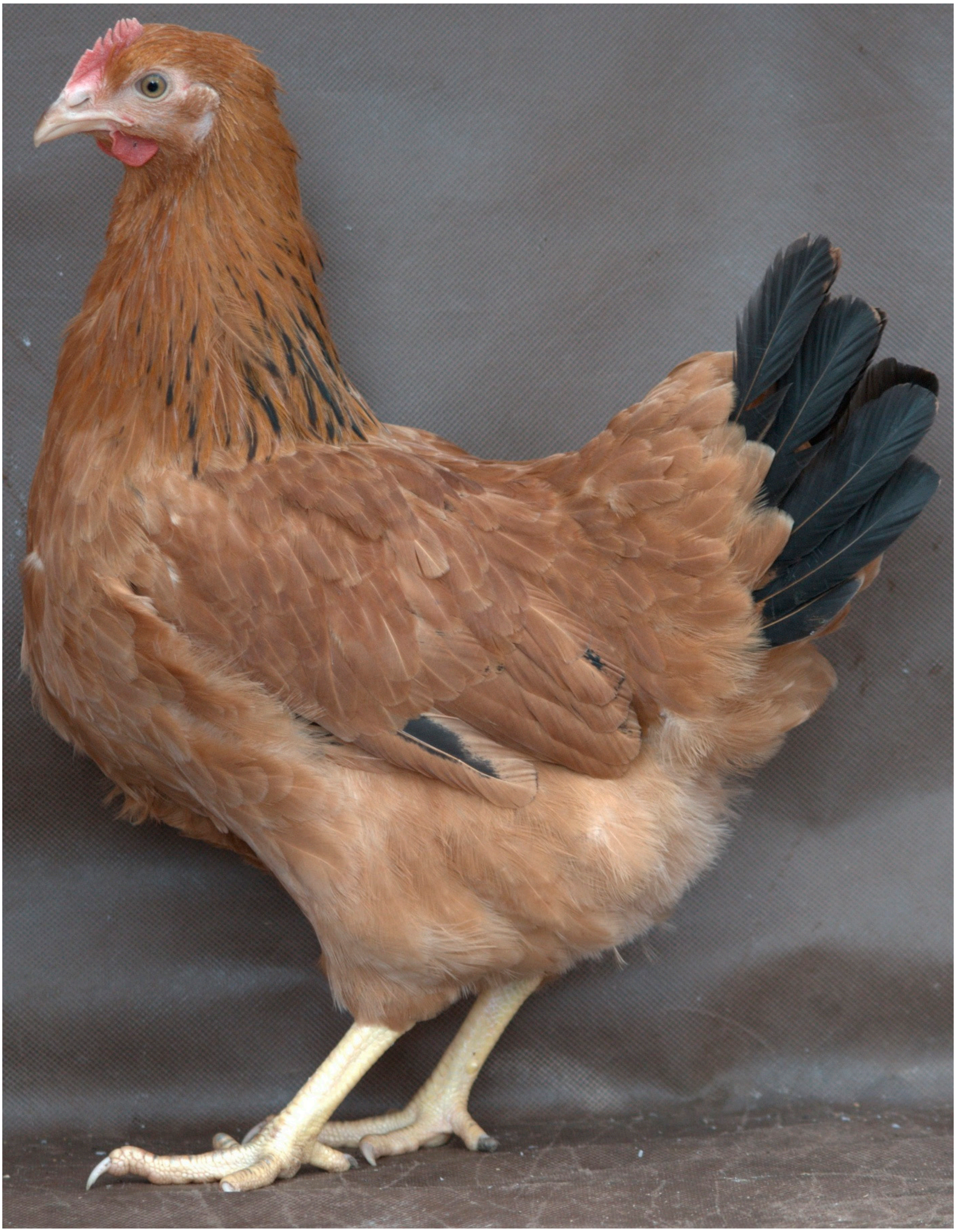

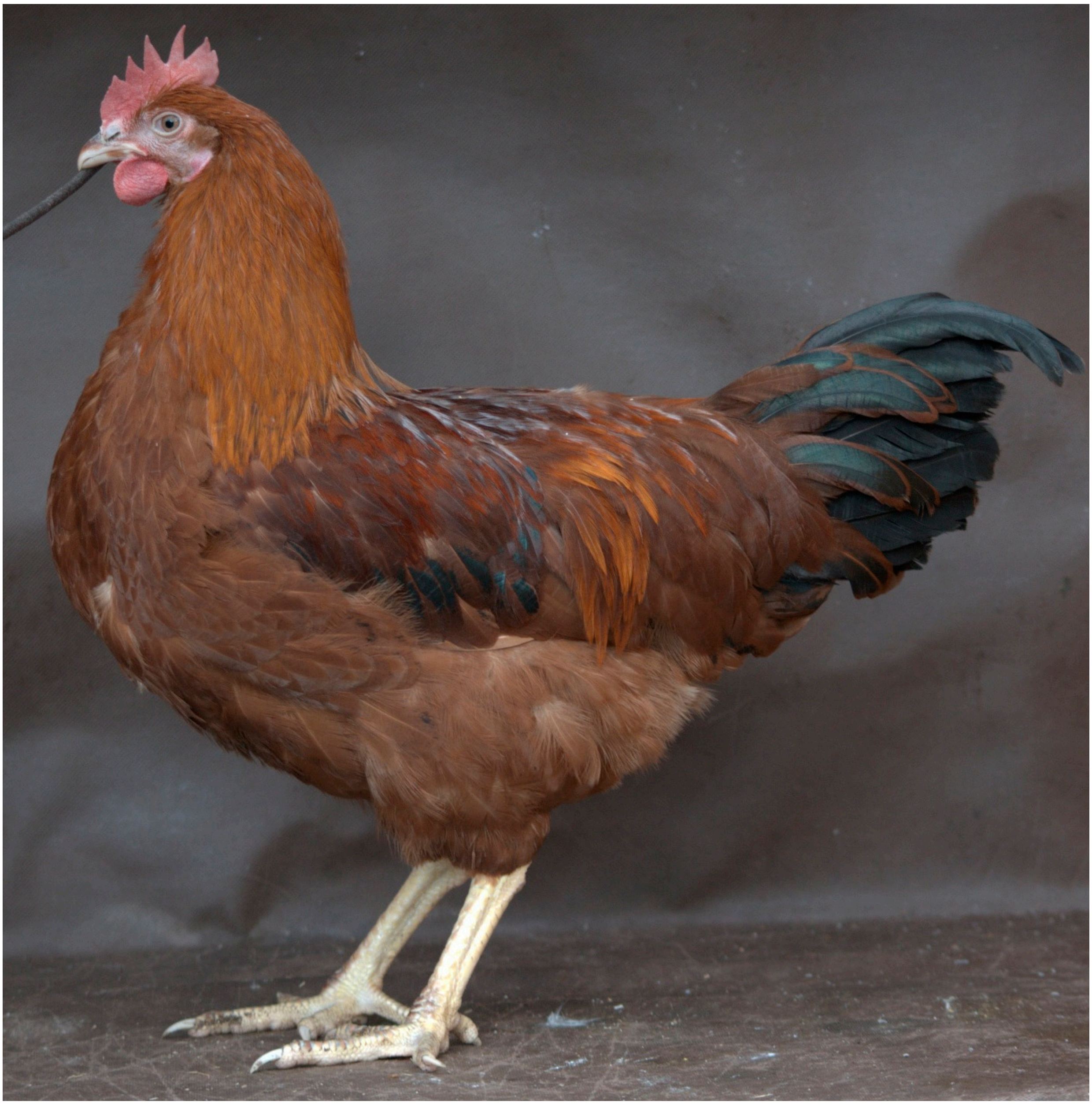

Figure S11. New-Hampshire chicken breed

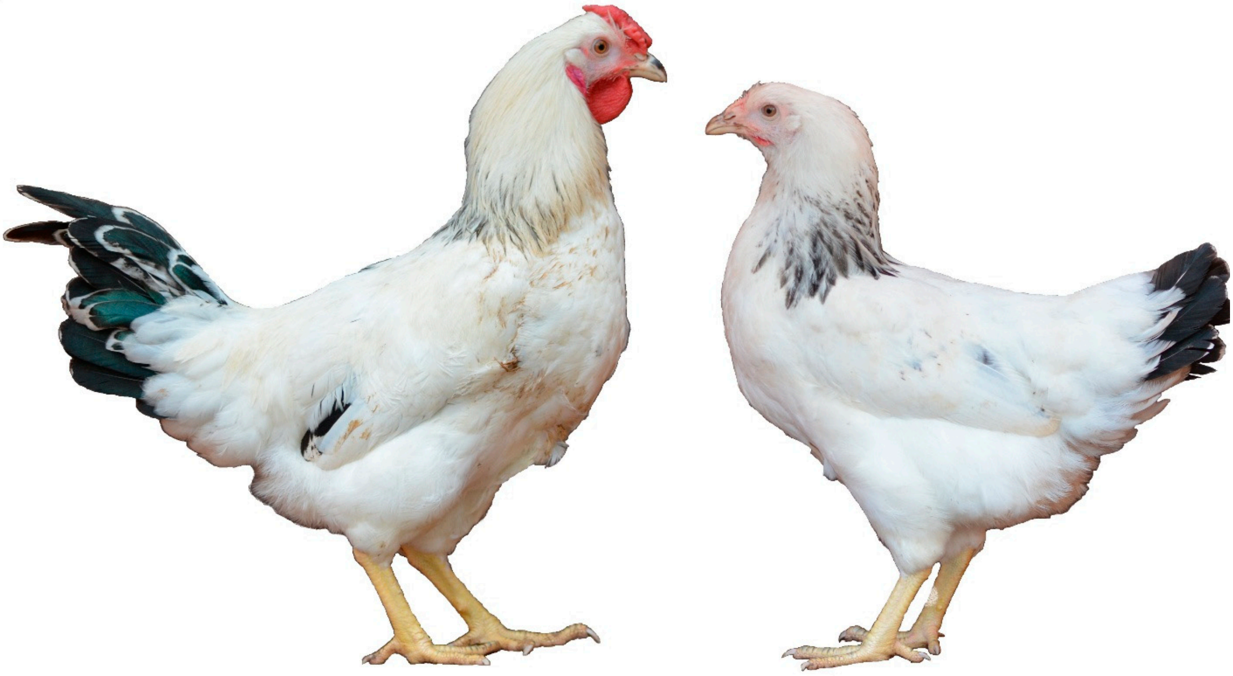

Figure S12. Pervomay chicken breed

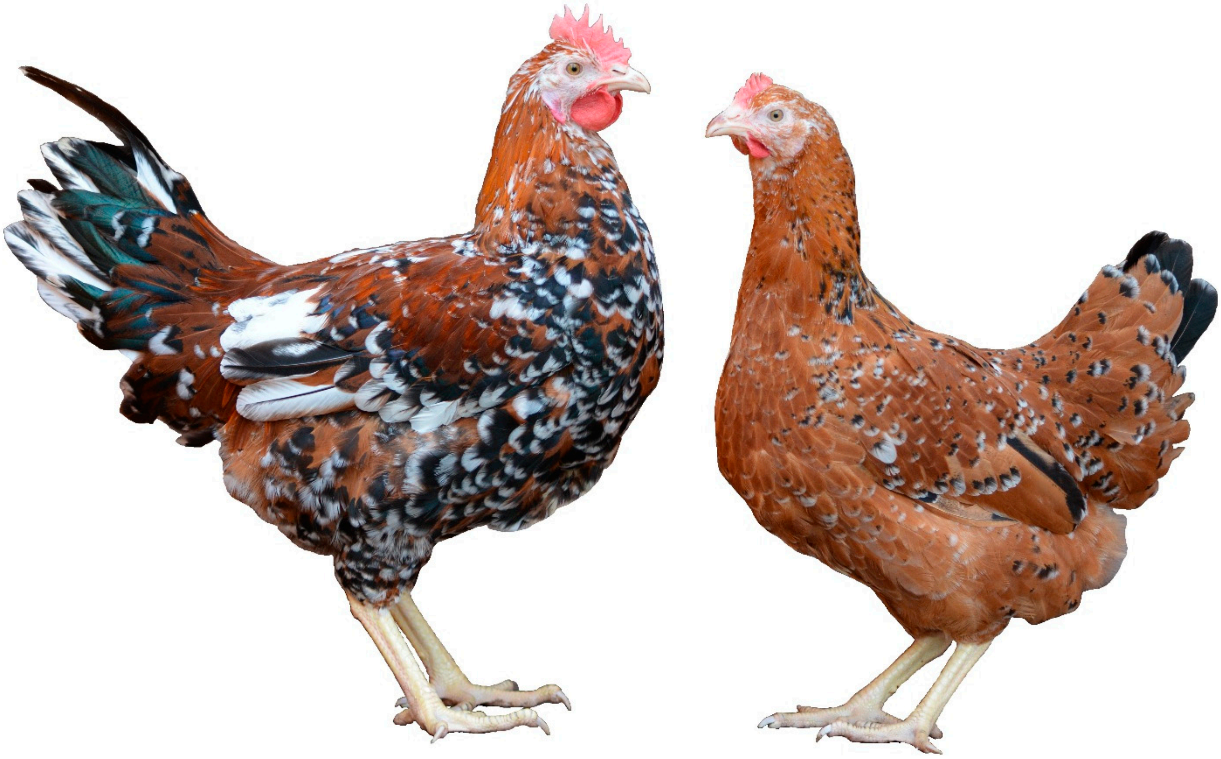

Figure S13. Leningrad Mille Fleur chicken breed

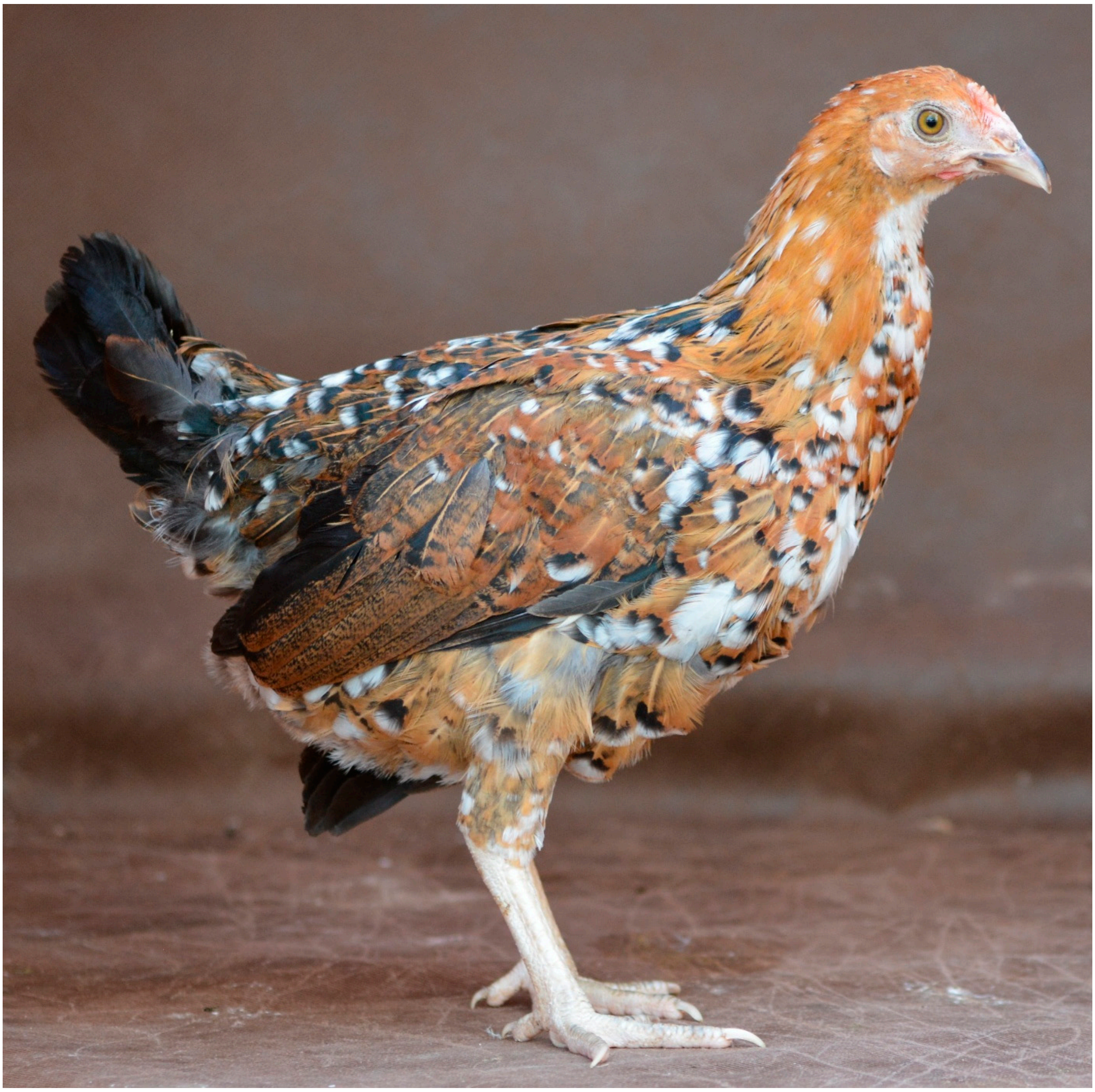

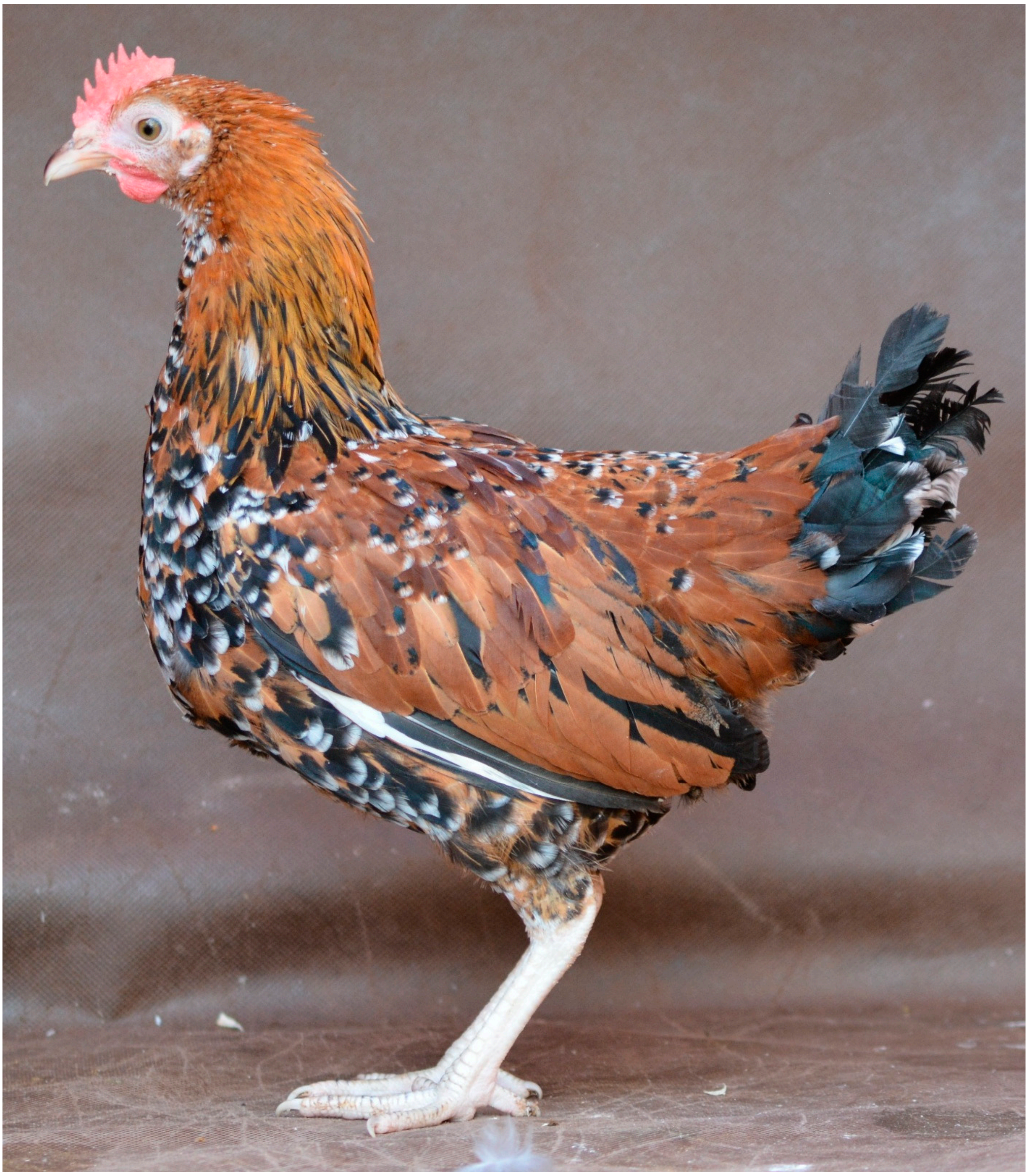

Figure S14. Bantam Mille Fleur chicken breed

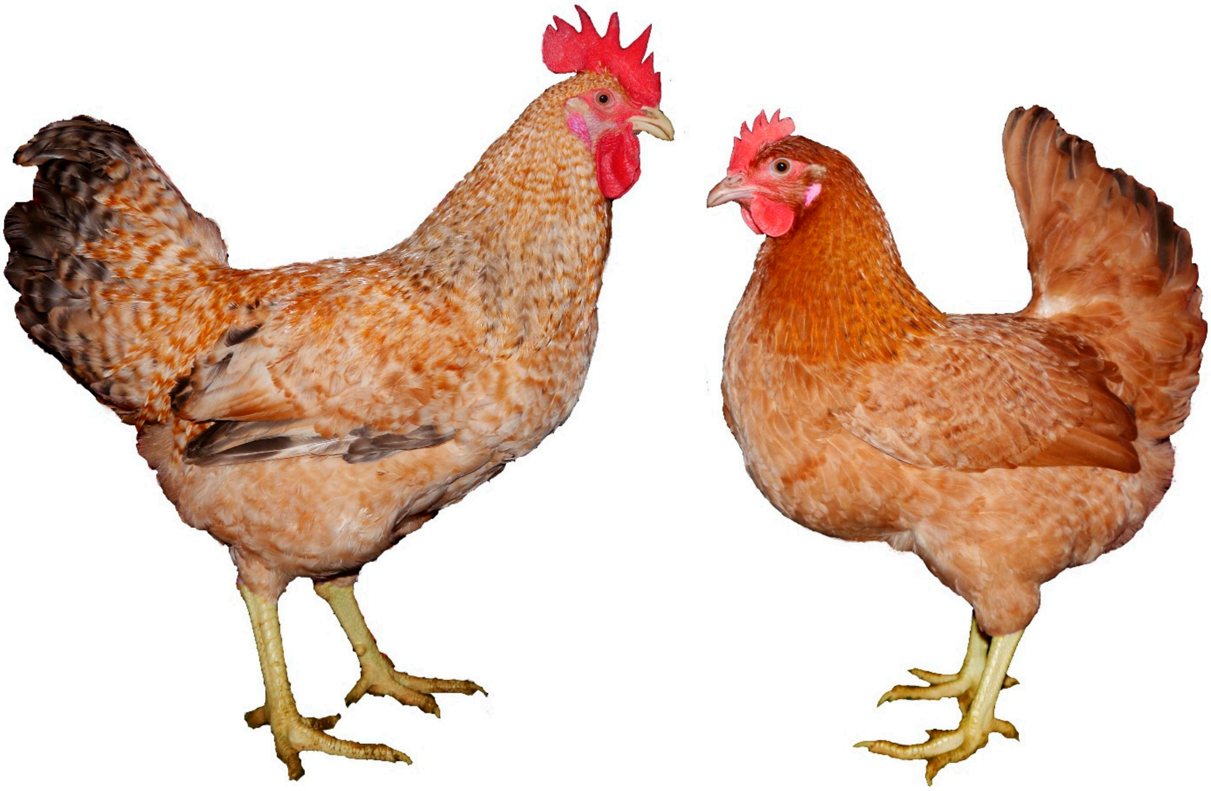

Figure S15. Tsarskoye Selo chicken breed

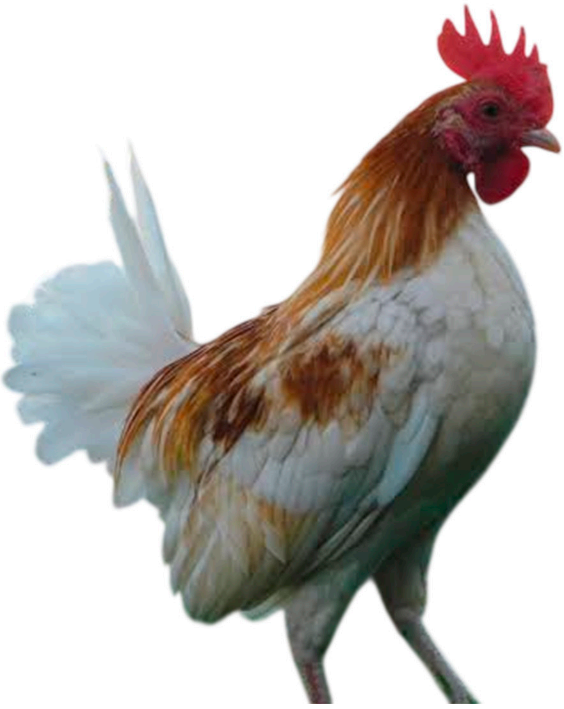

Figure S16. Red White-tailed Dwarf chicken breed

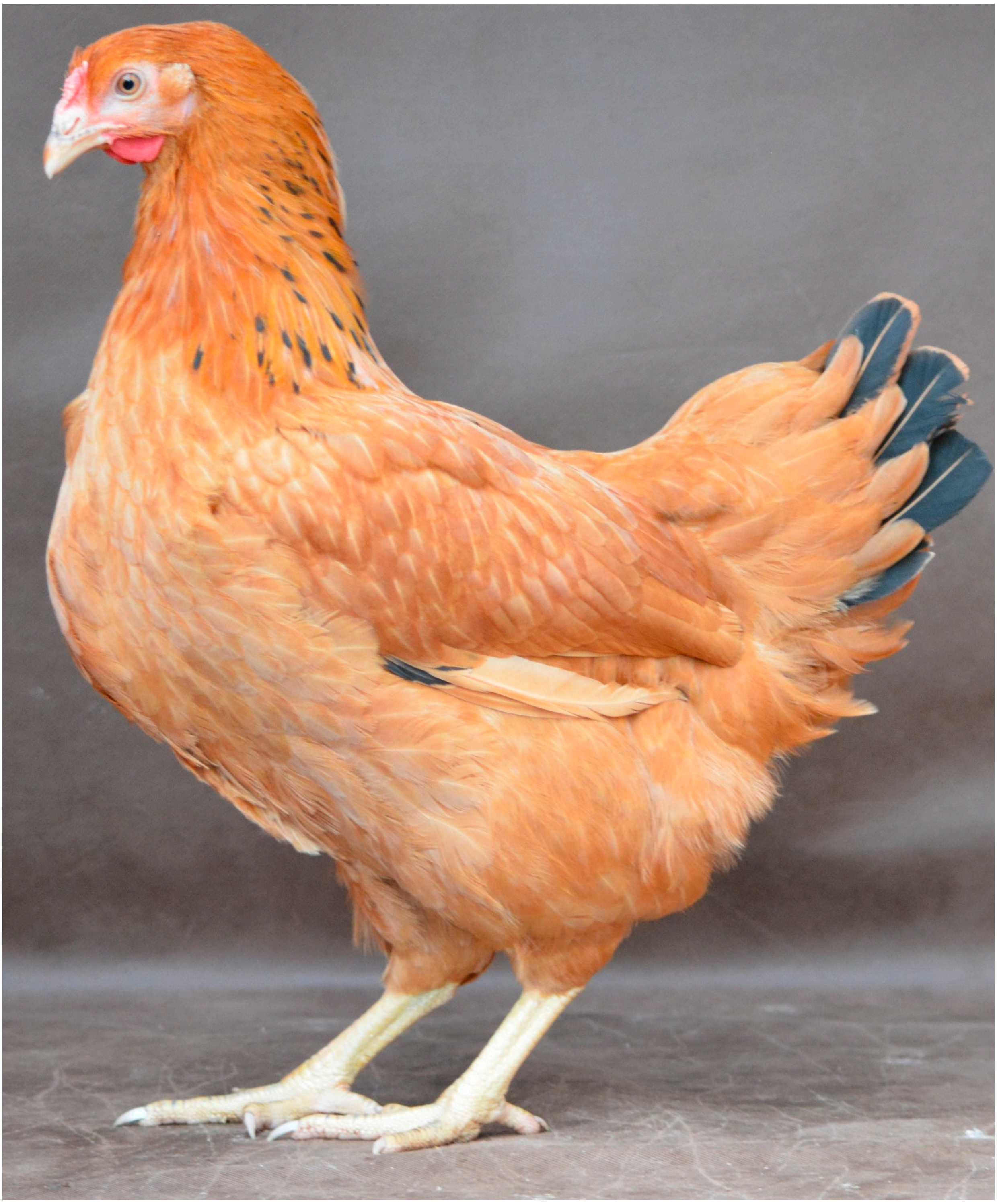

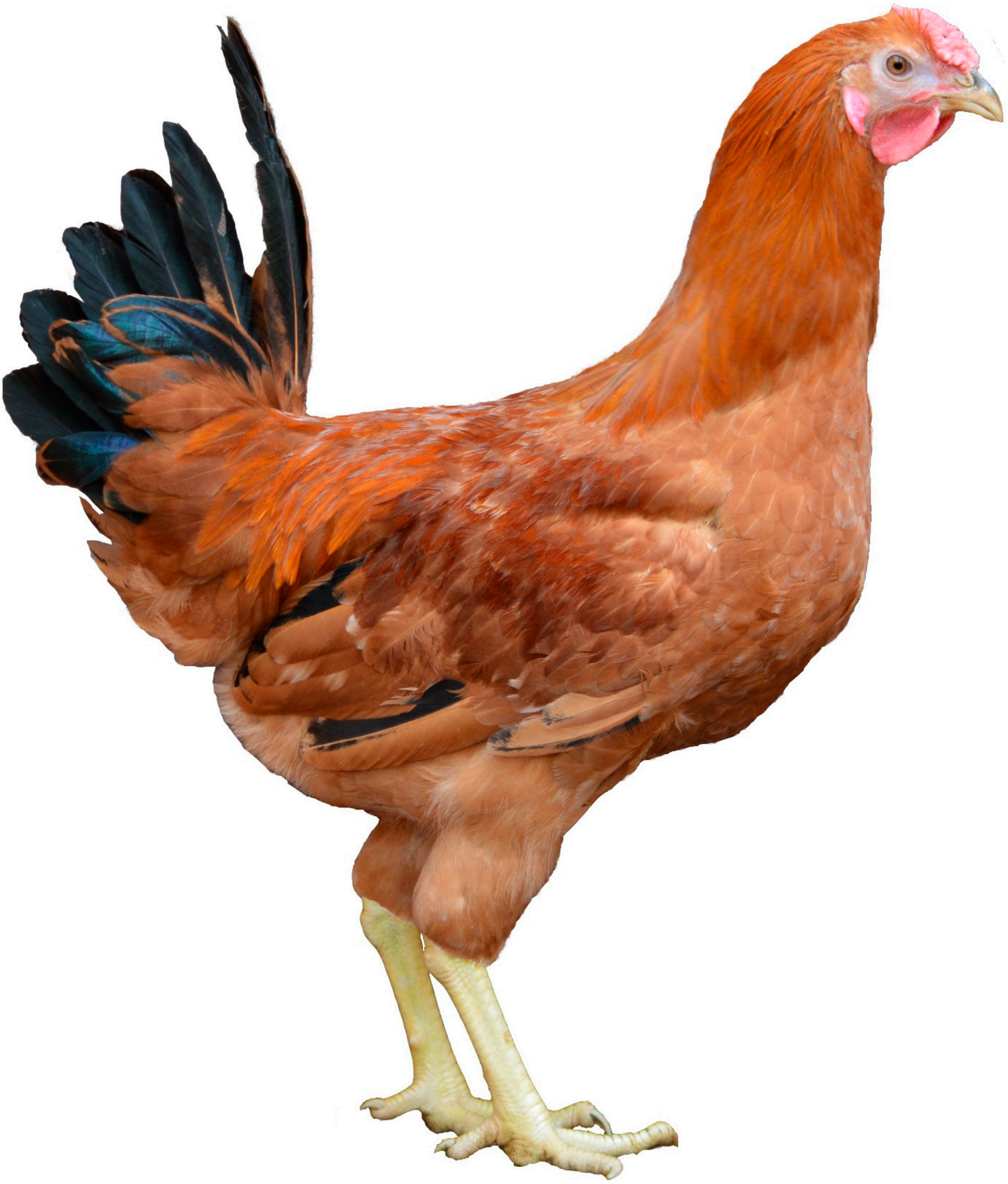

Figure S17. Poltava Clay chicken breed

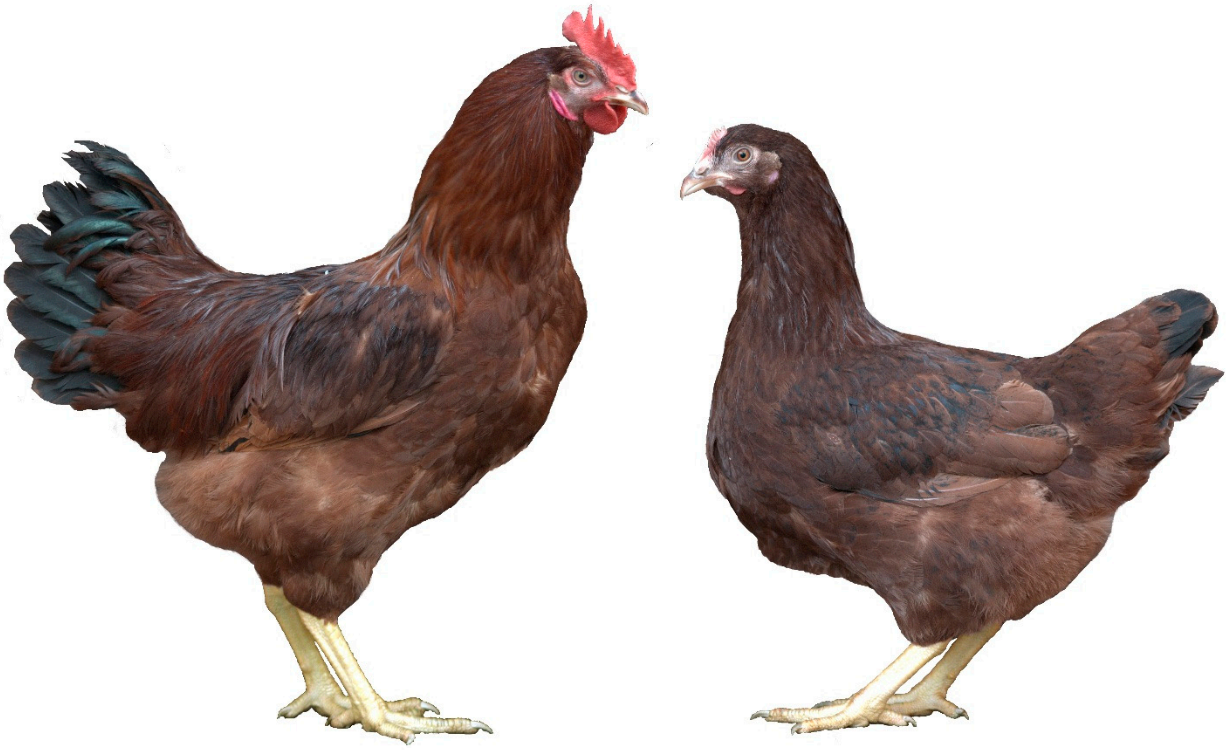

Figure S18. Rhode Island Red chicken breed
