# Supplementary figures and images for: Genomic Analysis of the Columbian Plumage Pattern in Various Chicken Breeds
Source: Animals (Basel). 2026 Jul 11;16(14):2153. doi: 10.3390/ani16142153 (PMC13404467; doi:10.3390/ani16142153)

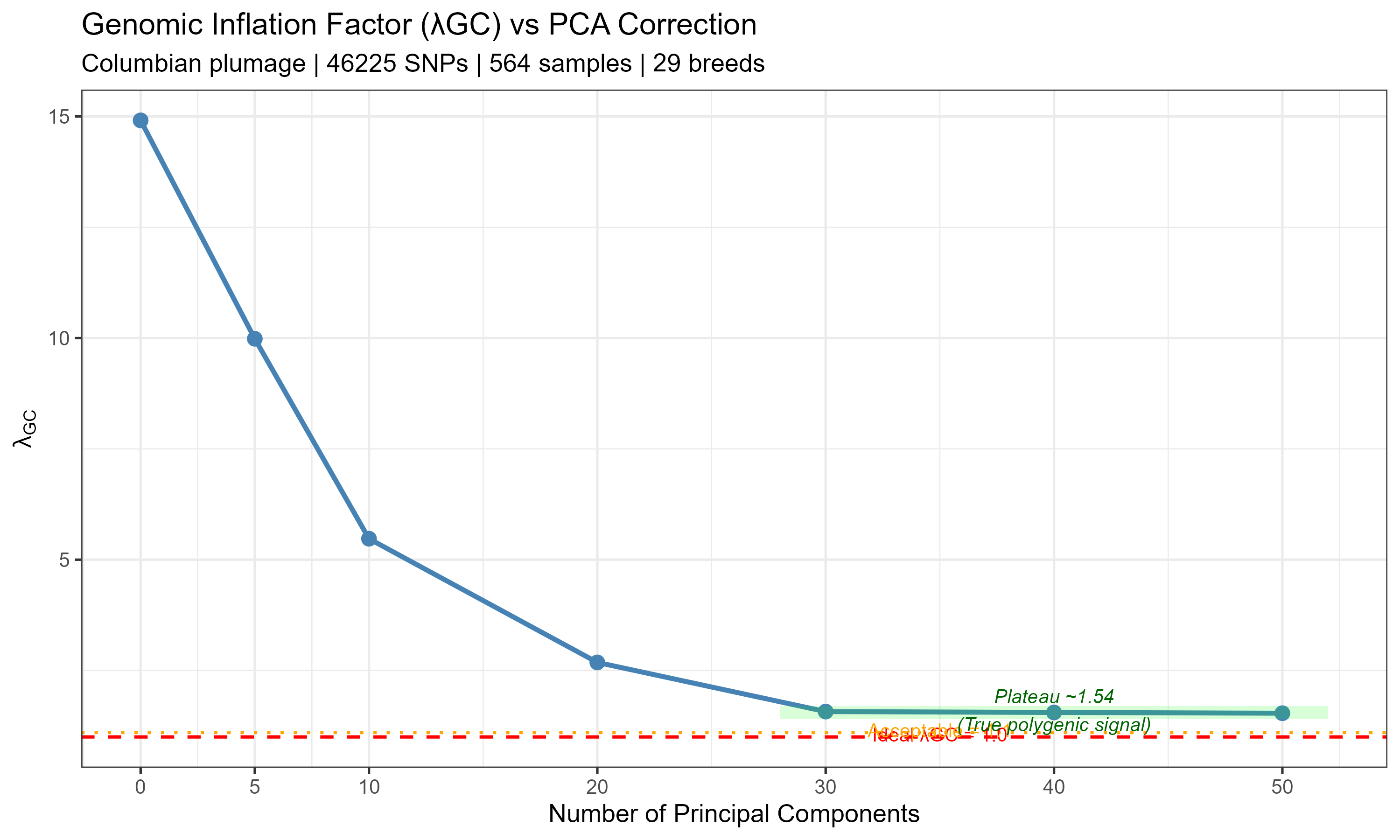

Supplement: Supplementary file 1 [file animals-16-02153-s001.zip › Figure S1. λGC vs PCs.png]

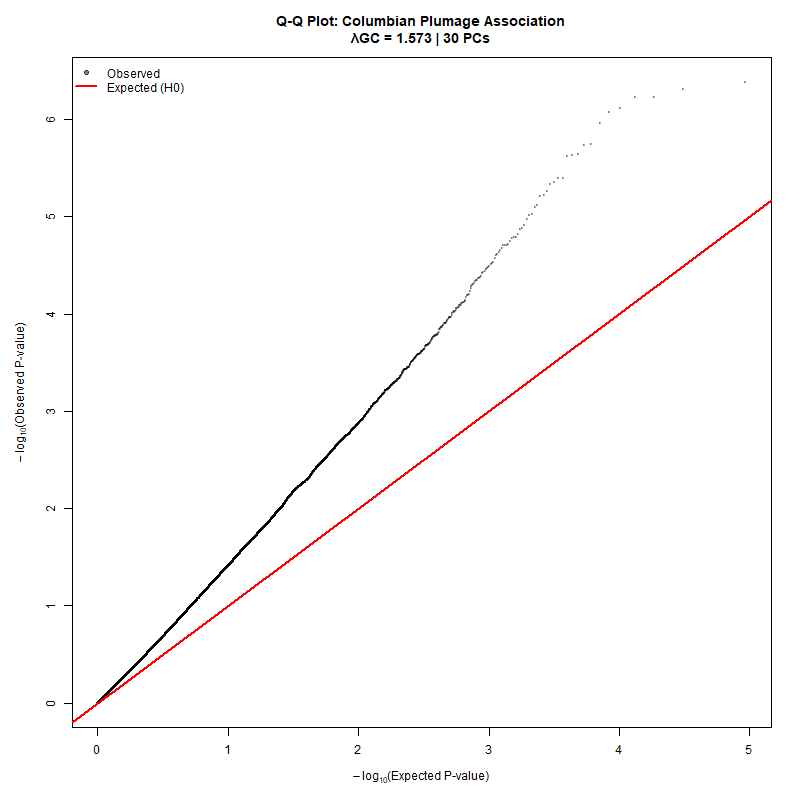

Supplement: Supplementary file 1 [file animals-16-02153-s001.zip › Figure S2. Q-Q plot (30 PCs, λGC = 1.57).png]

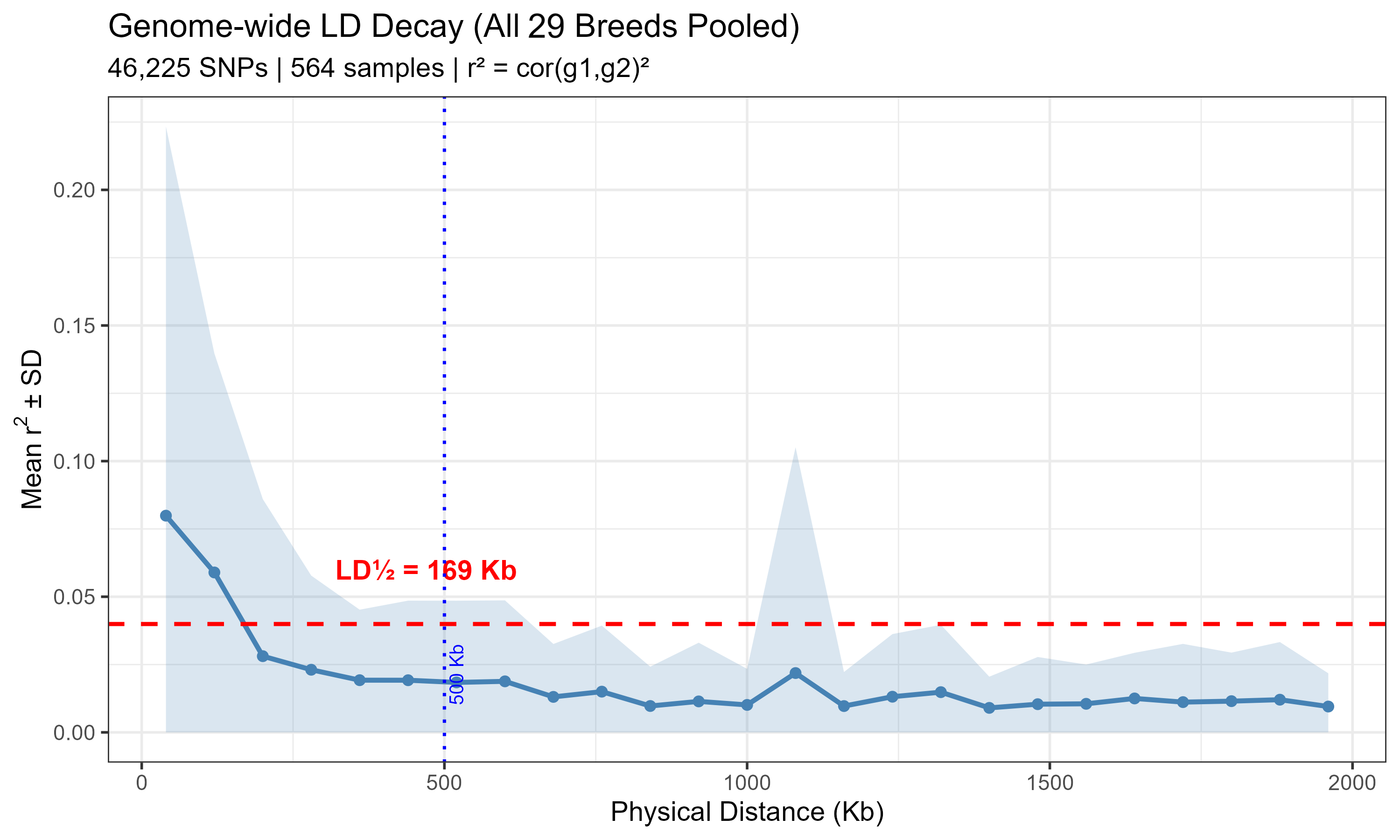

Supplement: Supplementary file 1 [file animals-16-02153-s001.zip › Figure S3. LD decay (pooled sample).png]

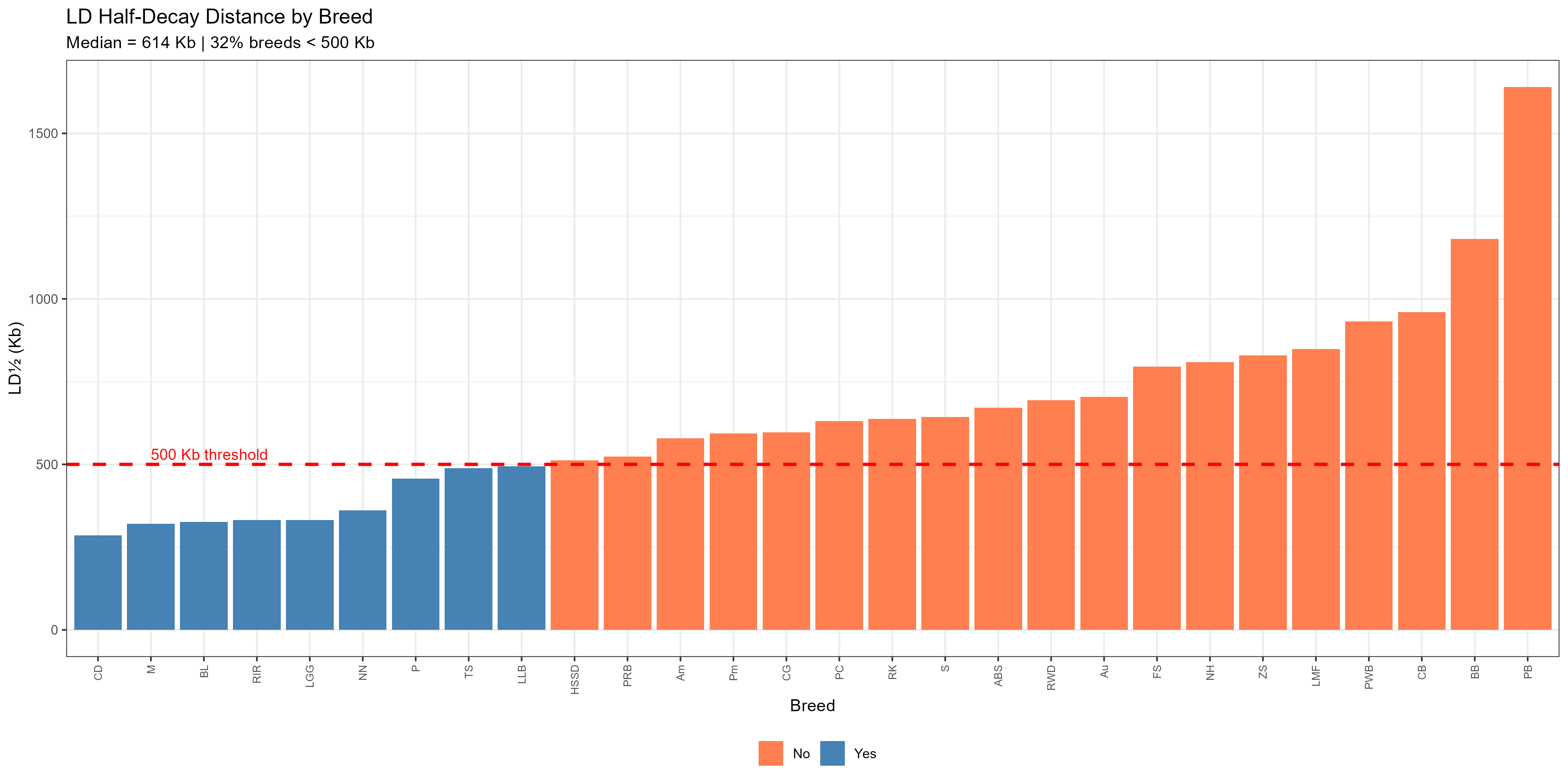

Supplement: Supplementary file 1 [file animals-16-02153-s001.zip › Figure S4. LD half-decay by breed.png]

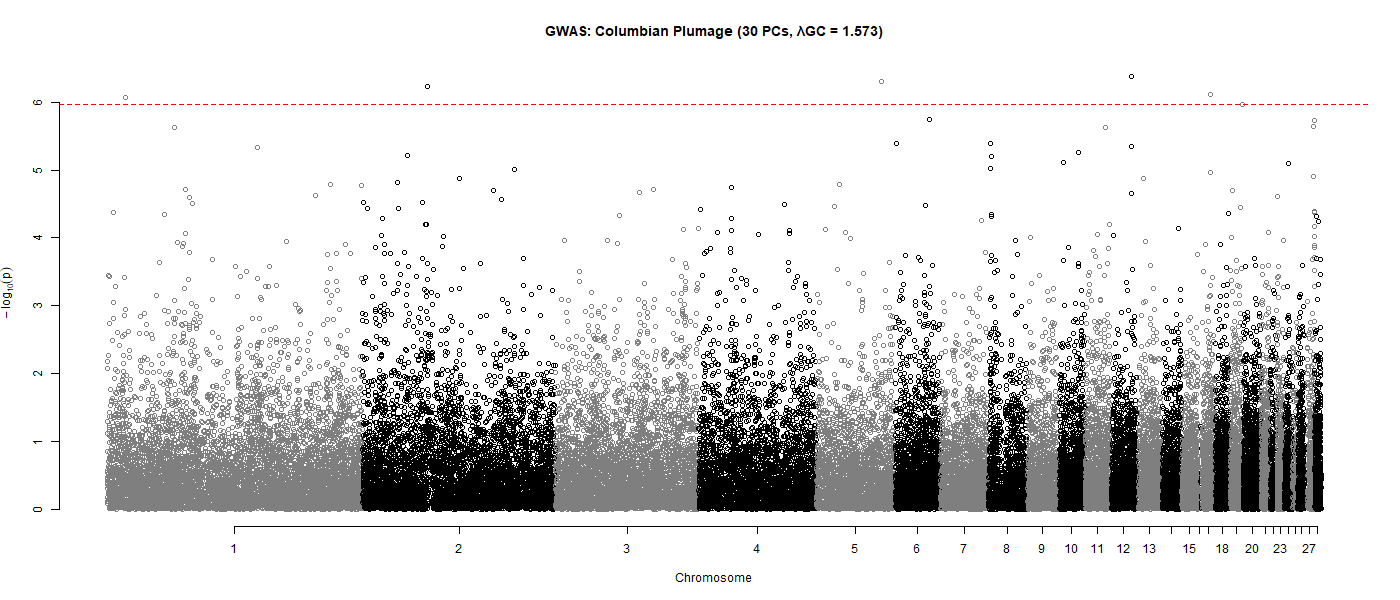

Supplement: Supplementary file 1 [file animals-16-02153-s001.zip › Figure S5. Manhattan plot after correction with 30 PCs.png]

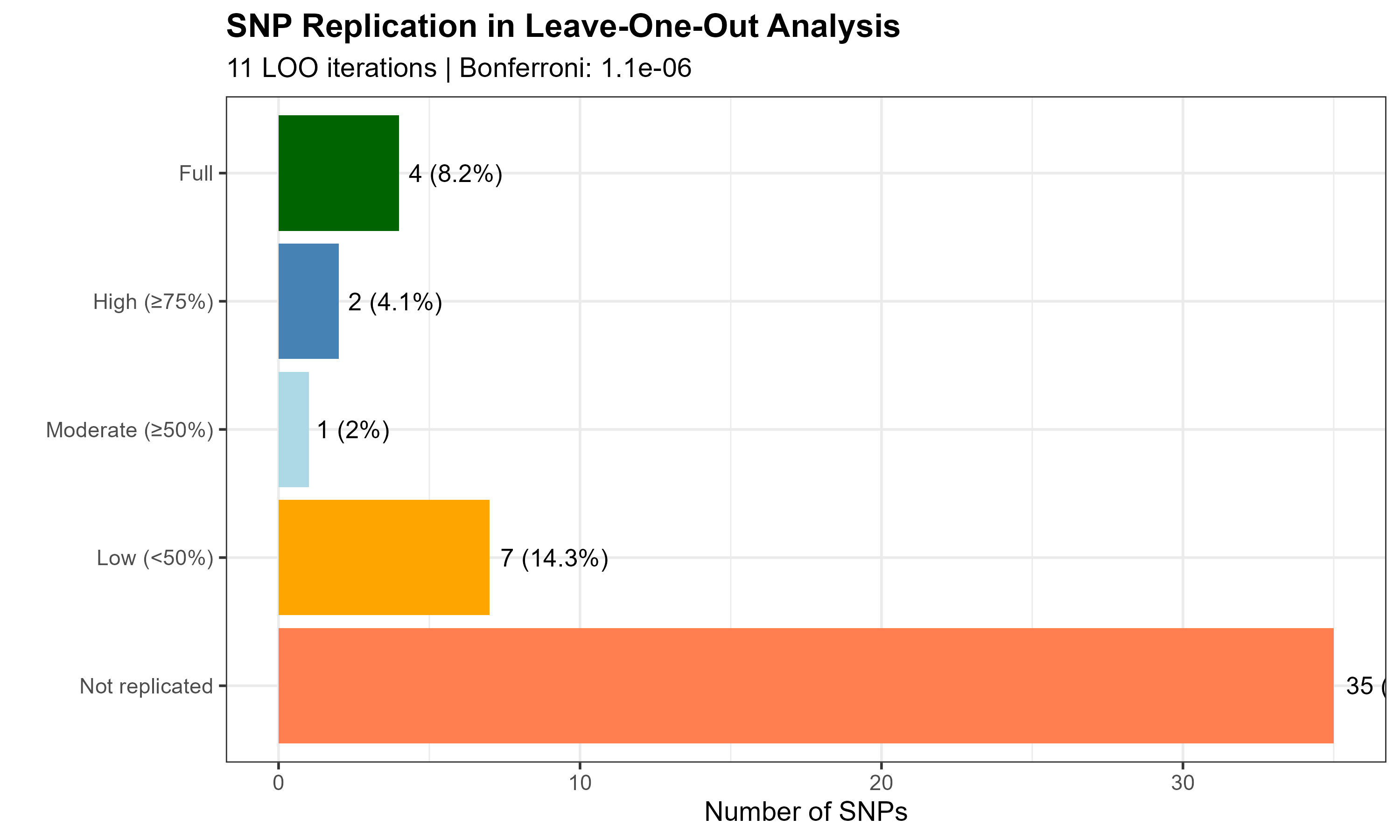

Supplement: Supplementary file 1 [file animals-16-02153-s001.zip › Figure S6. LOO replication categories.png]

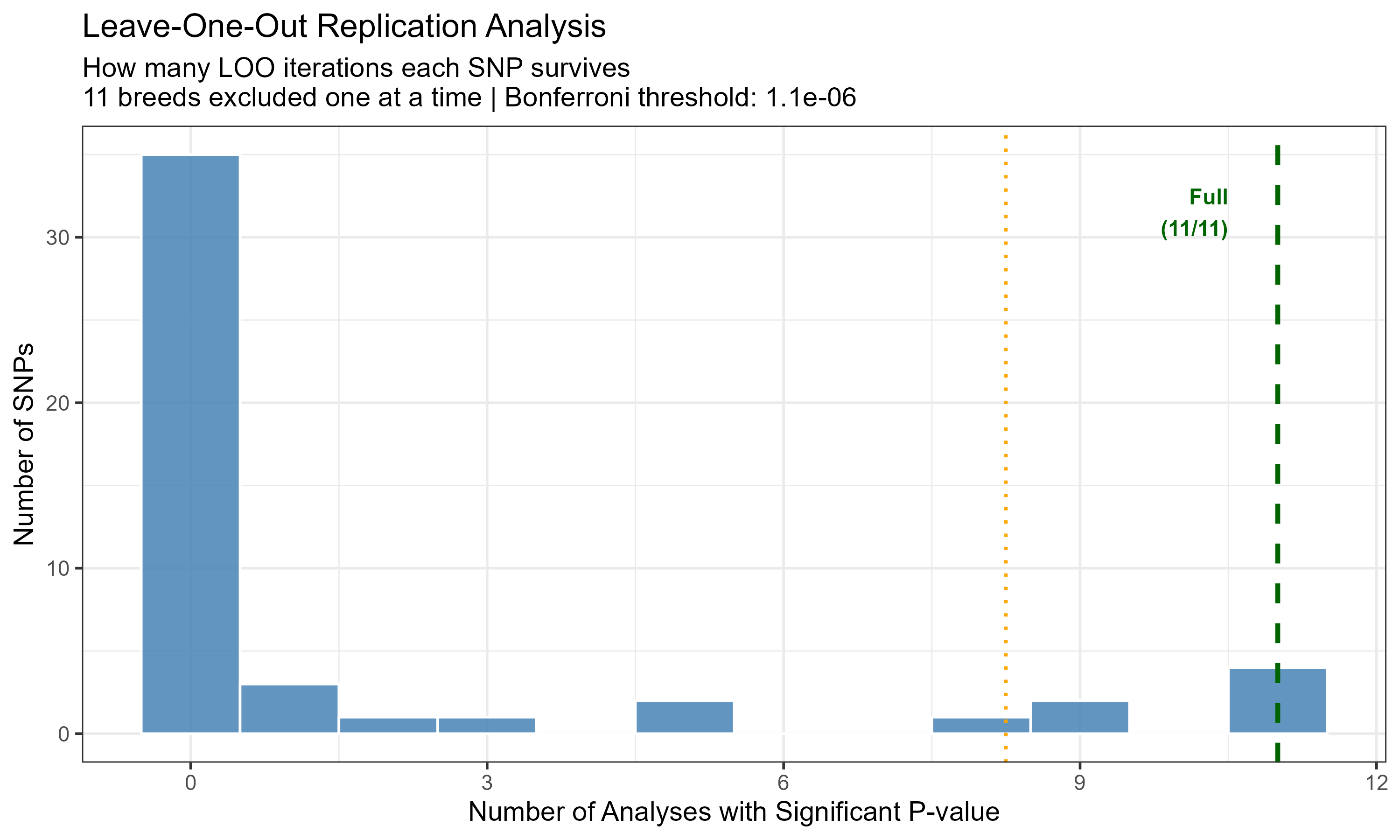

Supplement: Supplementary file 1 [file animals-16-02153-s001.zip › Figure S7. LOO replication histogram.png]
